# A new allele *PEL9* ^GG^ identified by genome-wide association study increases panicle elongation length in rice (*Oryza sativa* L.)

**DOI:** 10.3389/fpls.2023.1136549

**Published:** 2023-02-16

**Authors:** Xiaojing Dang, Chunyu Jing, Mengyuan Zhang, Xinru Li, Qing Xu, Changmin Hu, Yulong Li, Ying Zhang, Dezheng Wang, Delin Hong, Jianhua Jiang

**Affiliations:** ^1^ Institute of Rice Research, Anhui Academy of Agricultural Sciences, Hefei, China; ^2^ College of Agronomy, Anhui Agricultural University, Hefei, China; ^3^ Institute of Crop Research, Anhui Academy of Agricultural Sciences, Hefei, China; ^4^ National Key Laboratory of Crop Genetics & Germplasm Enhancement and Utilization, Nanjing Agricultural University, Nanjing, China

**Keywords:** genome-wide association study, hybrid rice seed production, male sterile line, panicle elongation length, quantitative trait loci, rice

## Abstract

Considering the male sterile line has the phenomenon of panicle enclosure, panicle elongation length (PEL) plays an important role in hybrid rice seed production. However, the molecular mechanism underlying this process is poorly understood. In this study, we investigated the PEL phenotypic values of 353 rice accessions across six environments, which shows abundant phenotypic variation. Combining the 1.3 million single-nucleotide polymorphisms, we performed a genome-wide association study on PEL. Three quantitative trait loci (QTL) *qPEL4*, *qPEL6*, and *qPEL9* were identified as significantly associated with PEL, of which *qPEL4* and *qPEL6* were previously reported QTLs and *qPEL9* was novel. One causal gene locus, *PEL9*, was identified and validated. The PEL of accessions carrying allele *PEL9* ^GG^ was significantly longer than that of those carrying allele *PEL9* ^TT^. We also demonstrated that the outcrossing rate of female parents carrying allele *PEL9* ^GG^ increased by 14.81% compared with that of the isogenic line carrying allele *PEL9* ^TT^ in an F_1_ hybrid seed production field. The allele frequency of *PEL9^GG^* increased gradually with an increase in latitude in the Northern Hemisphere. Our results should facilitate the improvement of the PEL of the female parent of hybrid rice.

## Introduction

Rice (*Oryza sativa* L.) provides the staple food for more than half of the world’s population and plays a vital role in global food security. To face the increasing challenges of food security caused by the increasing global population and decreasing arable land, it is an inevitable choice to increase yield per unit area. Utilization of heterosis is one of the most effective ways to increase yield per unit area. Hybrid rice has been planted on a commercial scale in China since 1976 and accounts for more than half of the total area of rice planted each year since 1985. Hybrid rice requires the annual production of F_1_ hybrid seeds. In the process of hybrid rice F_1_ seed production, the panicle enclosure of the female sterile line is an important factor limiting the yield of hybrid F_1_ seed production (Shen et al., 1987; Yang et al., 2002). The male sterile lines and female parent lines have different degrees of panicle enclosure, which showed 30%–60% of the panicle to be enclosed by a flag leaf sheath and the panicle neck shortened. Generally, spraying exogenous gibberellin acid 3 (GA_3_) on the female sterile line at the initial heading stage in the F_1_ seed production field can improve the panicle enclosure. However, spraying a large amount of GA_3_ has the shortcomings of increasing seed production costs, polluting the environment, and increasing the occurrence of rice kernel smut (Brooks et al., 2009; Chen et al., 2016). Therefore, it is an important goal for hybrid rice breeding to genetically eliminate the panicle enclosure of cytoplasmic male-sterile (CMS) lines.

**Figure 1:**
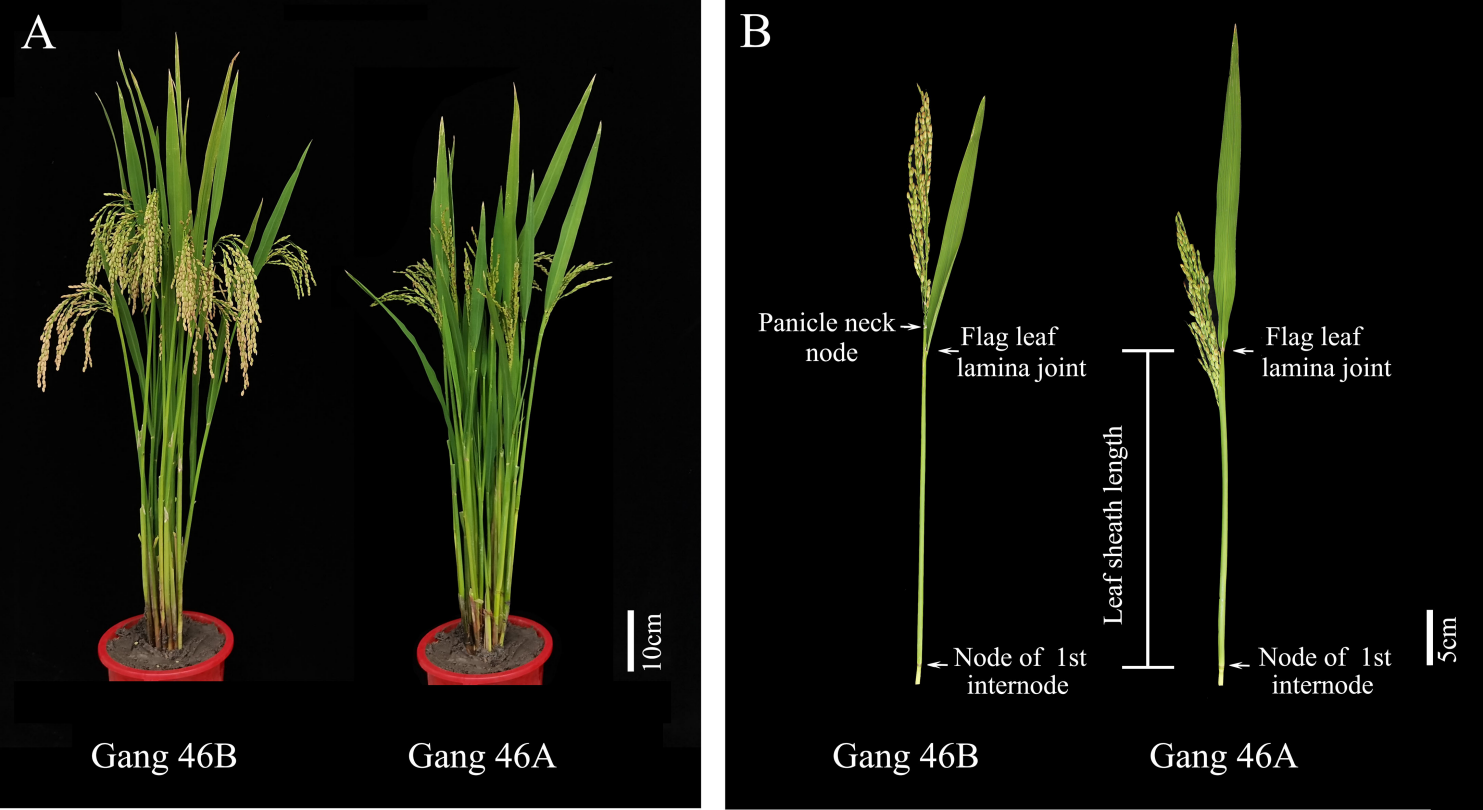
Display the plants, panicle, and uppermost internode of Gang 46B and Gang 46A. **(A)** The plant morphology of Gang 46B and Gang 46A. Scale bar, 10cm. **(B)** Display the panicle morphology and uppermost internode of Gang 46B and Gang 46A. Scale bar, 5 cm.

The phenomenon of panicle enclosure in CMS line was caused by sterile cytoplasm, not by the nucleus, because panicle enclosure did not exist in the maintainer line. The leaf sheath of the flag leaf in the CMS line and maintainer line has the same length (**Figure 1**), so the sterile cytoplasm caused the shortening the uppermost internode, which led to panicle enclosure. Only when the maintainer line with the longer panicle elongation length (PEL) is transformed into the CMS line by continuous backcrossing will there be no panicle enclosure.

PEL is a quantitative trait controlled by multiple genes in rice (Han et al., 2005; Jing et al., 2015). At first, Rutger and Carnahan (1981) found a long panicle neck mutant in the F_3_ population from a cross between two japonica rice cultivars and named the mutant *elongated uppermost internode* (*eui*). Yang et al. (2001) reported that two *eui* genes named *eui1* and *eui2* were obtained from the indica rice cultivar Xieqingzao B by nuclear mutagenesis. The mutant with panicle enclosure could be obtained by natural variation, physical and chemical mutagenesis, and tissue culture; until now, 11 mutants (*A864*, *shp1*, *shp2*, *shp3*, *shp4*, *shp5*, *shp6*, *M893*, SUI-family (*sui1*, *sui2*, *sui3*, and *sui4*), *esp1*, and *esp2*) have been reported (Liu and Luo, 2006; Zhu, 2006; Guan et al., 2011; Zhu et al., 2011; Yin et al., 2013; Ma et al., 2016). Among them, *SHP5* and *SHP6* were detected on chromosomes 4 and 2, respectively (Zhu, 2006). *esp1* was located on chromosome 11 (Zhuo, 2010). *SUI2* was finely mapped within the 110 kb region of the long arm of chromosome 4 and the candidate gene predicted was *LOC_Os04g39430* (Sun et al., 2020). *esp2* was finely mapped within the 14 kb region of the long arm of chromosome 1 and the candidate gene predicted was *LOC_Os01g02890* (Guan et al., 2011).

To our knowledge, 56 quantitative trait loci (QTLs) controlling PEL have been identified and distributed on 12 chromosomes of rice (Yamamoto et al., 2001; Hittalmani et al., 2002; Zhu, 2003; Zhu et al., 2006; Qiao et al., 2007; Xiao et al., 2008; Yang et al., 2009; Yang et al., 2011; Yin et al., 2013; Jing et al., 2015; Zhao et al., 2015; Gao et al., 2016; Dang et al., 2017). Four genes, *EUI2*, *EUI1*, *SUI1*, and *Hox12*, have been cloned to control PEL. *Eui2* encodes an epoxide hydrolase, which is involved in the dehydrogenation of reactive GA. In the process of dehydrogenation, the epoxide hydrolase loses its activity, which affects the elongation of the uppermost internode (Zhu, 2003). *EUI1* encodes a cytochrome P450 monooxygenase, *CYP714D1. CYP714D1* catalyzes the 16a,17-epoxidation of non-13-hydroxylated GAs to reduce the biological activity of GA_4_ to regulate the elongation of the uppermost internode in rice (Luo et al., 2006; Zhu et al., 2006). *SUI1* encodes phosphatidylserine synthase *OsPSS-1. OsPSS-1* controls cell elongation especially in the uppermost internode by regulating exocytosis in rice (Zhu et al., 2011; Yin et al., 2013; Ma et al., 2016). *Hox12* regulates panicle exertion by directly modulating the expression of *EUI1* (Gao et al., 2016). However, these genes/QTLs are far from satisfying the need for genetic improvement in PEL. Therefore, it is necessary to identify more favorable alleles for the PEL trait to enhance the yield of hybrid seed production in rice.

In this study, we conducted a genome-wide association study (GWAS) for the PEL trait using single nucleotide polymorphism (SNP) loci from 353 rice accessions and detected three QTLs for PEL. We further validated *panicle elongation length 9* (*PEL9*), coding for a flavonol synthase, as a causal gene for QTL *qPEL9* associated with PEL by gene-based association, transgenic overexpression testing, and field F_1_ hybrid seed production. The elite alleles of *PEL9* identified in this study will be facilitated for rice breeding to improve the PEL of the parents of hybrid rice.

## Materials and methods

### Plant materials and field planting

A natural population consisting of 353 rice accessions (Dang et al., 2020) from different geographical latitudes was selected (**Table S1**). These accessions were grown from May to October across six different environments over 3 years (2019–2021) and two locations. The two locations were Nanjing (32°07′N, 118°64′E) in Jiangsu Province and Hefei (31°86′N, 117°28′E) in Anhui Province. We named 2019 in Nanjing as environment 1 (E1), 2020 in Nanjing as environment 2 (E2), 2021 in Nanjing as environment 3 (E3), 2019 in Hefei as environment 4 (E4), 2020 in Hefei as environment 5 (E5), and 2021 in Hefei as environment 6 (E6). In each environment, we performed the field planting of 353 accessions with two replicates in a completely randomized block design. Each accession was planted with 40 plants in four rows with a spacing of 20 cm × 17 cm. All field trials followed routine agricultural management.

### Phenotypic measurement

Five plants in the middle row of each accession were selected to measure the PEL of the main stem panicle. The distance between the panicle base node and the collar of the flag leaf was measured as PEL. If the panicle base node was below the collar of the flag leaf, we recorded the PEL as a negative value. If the panicle base node was above the collar of the flag leaf, we recorded the PEL as a positive value. If the former and the latter were in the same position, we recorded the PEL as zero. We used the average value of the five measurements for each plot to further analyze.

### Genome-wide association study

The 1,326,094 rice SNP markers were used for the GWAS as described by Dang et al. (2020). For these SNPs, the nucleotide variation missing rates were lower than 0.25 and the minor allele frequency (MAF) was higher than 0.05. The GWAS was conducted using a compressed mixed linear model (MLM) program by GAPIT v. 2.12 (Lipka et al., 2012). The software of the qqman package in R (Turner, 2018) was used to generate the Manhattan plot and Q-Q plot. Based on the Benjamini and Hochberg (1995) correction method, we calculated the false discovery rate (FDR) for significant associations. We selected 1.0 × 10^−5^ as the significant threshold. We conducted the linkage disequilibrium (LD) analysis using the software Haploview 4.2 (Barrett et al., 2005) to define LD blocks surrounding significant SNPs. We constructed LD heatmaps surrounding peaks using the R package “LDheatmap” (Shin et al., 2006). According to the method described by Yang et al. (2014) and Huang et al. (2021), when more than three association loci exceed the threshold line of the *P*-value with a clear peak-like signal, we consider the region exists a QTL. The haplotypes comprising at least 20 investigated varieties were selected for comparative analysis.

### Candidate gene analysis

We predicted candidate genes within a 200 kb genomic region based on position information from the MSU7 database (Rice Genome Browser: http://rice.plantbiology.msu.edu). The 200 kb genomic region was ±100 kb of the significant leading SNP of the QTL following the description by Lu et al. (2016). We analyzed the SNP types for each candidate gene located in the candidate region. Gene annotation and gene expression levels for the candidate genes were analyzed to further select the causal gene.

### RNA extraction and quantitative real-time PCR

Total RNA was extracted from the internode directly below the young panicle at development stages 5–8 (the criterion of the corresponding development stage described by Itoh et al., 2005) using the ultrapure RNA kit (OMEGA BIO-TEK, https://www.omegabiotek.com). The uppermost internode was sampled from the three accessions with a shorter PEL and the three accessions with a longer PEL. The HiScript II Reverse Transcriptase SuperMix (Vazyme Biotech Co., Ltd., Nanjing; http://www.vazyme.com) was used to perform first-strand cDNA synthesis by reverse transcription from 1 µg of RNA. We used the 18S rRNA gene as an internal control. We used SYBR Green (Vazyme Biotech Co., Ltd., Nanjing; http://www.vazyme.com) to conduct real-time quantitative RT-PCR in a 96-well thermocycler (Roche Applied Science LightCycler 480). The cycling conditions were as follows: 1) denaturation, 95°C/5 min; 2) amplification and quantification program with a single fluorescence measurement including 40 cycles, 95°C/10 min, 60°C/30 s, and 72°C/60 s; 3) melting curve, 60°C–95°C, with a heating rate of 0.1°C/1 s and continuous fluorescence measurement; 4) cooling, 40°C. For each sample, we performed three biological replicates. The primer sequences used for qRT-PCR are listed in **Table S2**. We used the comparative *C_T_* method (Livak and Schmittgen, 2001) to calculate the transcript levels of gene expression.

### Generation of *LOC_Os09g18390* transgenic plants

The coding sequence of *LOC_Os09g18390* was PCR amplified from Nipponbare (carrying the GG allele) and Nongxiang 25 (carrying the TT allele), respectively. The PCR products were cloned into the pBWA(V)HS vector. The primer sets used for PCR are listed in **Table S2**. The vectors *LOC_Os0918390^GG^* and *LOC_Os0918390^TT^* were introduced into *Agrobacterium tumefaciens* (EHA105) and transferred into Nipponbare, respectively. The vector *LOC_Os0918390^GG^* with EHA105 was also transferred to 7001S. We transformed the corresponding empty vector into Nipponbare as a control. We obtained 30 independent T_1_ seedlings, which were grown in a paddy field under natural conditions. We harvested the T_2_ seeds from T_1_ plants at the maturity stage and grew them in the paddy field in the next rice growing season (May to October). We detected the three allele genotypes (GG, GT, and TT) on the *LOC_Os09g18390* locus at the tillering stage using the primers listed in **Table S2**. We measured the PEL phenotype in the *LOC_Os0918390^GG^* and *LOC_Os0918390^TT^* plants after the heading dates.

### Pollen fertility observation

The mature pollen grains of varieties R1219, 7001S*^PEL9GG^* and 7001S*^PEL9TT^* were stained using a 1% I2-KI water solution. We examined them with a light microscope (Olympus BH-2) at ×100 magnification.

### F_1_ hybrid seed production potential evaluation for the *PEL9* alleles in the paddy field

To evaluate the potential of the *PEL9* gene in the production of F_1_ hybrid rice seeds, we performed an actual F_1_ seed production experiment using isogenic lines 7001S*^PEL9TT^* (short PEL) and 7001S*^PEL9GG^* (long PEL) as the female parents and variety R1219 as the common male parent in the paddy fields. 7001S*^PEL9TT^* is a long day-sensitive male sterile line used in commercial F_1_ seed production in Eastern China, and 7001S*^PEL9GG^* is an isogenic line obtained in this study. The male and female parents were grown in a ratio of 2:8:2, i.e., four lines of R1219 plants were planted around eight lines of female plants. To ensure that the pollen of the female parents was sterile before artificial pollination at flowering time, we observed the fertility of the pollen of 7001S*^PEL9TT^* and 7001S*^PEL9GG^* under a light microscope. During pollen dispersal, artificial supplementary pollination was performed twice per day. Thirty days after artificial supplementary pollination, the seeds from the female plants were harvested individually. The potential of the *PEL9* allele for hybrid rice seed production was evaluated according to the weight of rice grains harvested from male sterile plants per 1 m^2^ of area.

## Results

### Phenotypic variation of the PEL trait

**Figure 2:**
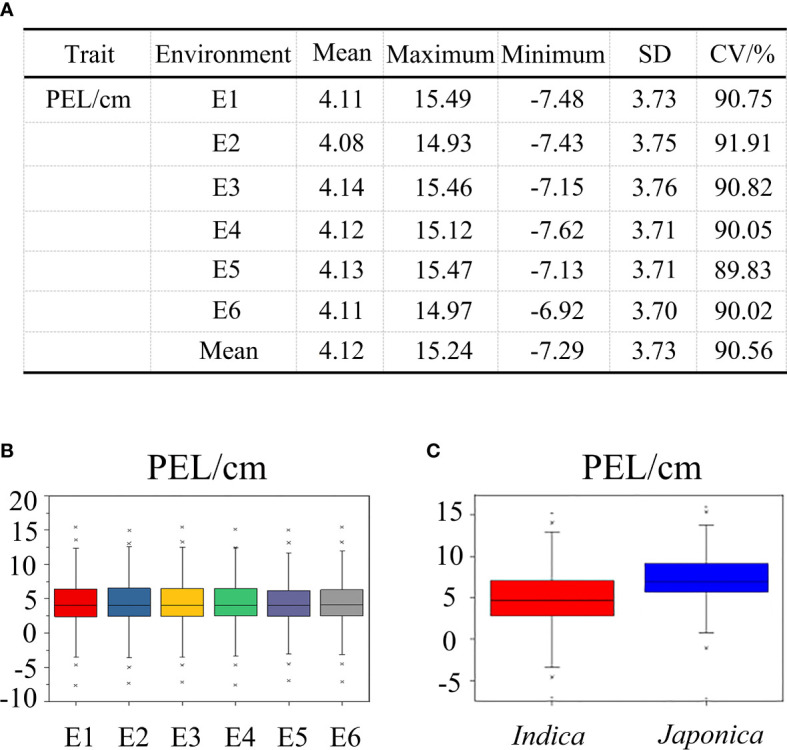
The phenotypic value description of PEL among 353 rice accessions at six environments. **(A)** Basic statistics of PEL in six environments. **(B)** Phenotypic value distributions in six environments. **(C)** Phenotypic value distributions of PEL in the indica and japonica subgroups. The box edges represent the upper and lower quantiles. The median value in black was shown in the middle of the box. Vertical lines represent the data from the lowest quantile to the top quantile. Asterisks indicate individuals falling outside the range of the whiskers.

In the 353 accessions, the mean value of PEL was calculated per environment, ranging from −7.62 ± 0.35 to 15.49 ± 0.24 cm, with coefficients of variation across the six environments ranging from 89.83% to 91.91% (**Figure 2A**). The PEL trait had no significant change among six environments, indicating that this trait was less influenced by the environment (**Figure 2B**). The results mentioned above indicated that a wide range of phenotypic variation existed in the natural populations studied. Compared with *japonica* rice, the *indica* rice population had lower values for the PEL trait (**Figure 2C**). The results of the joint analysis of variance for PEL showed that there was a significant difference among genotypes and no significant differences among environments, suggesting that the abundant phenotypic variation of PEL was mainly attributable to variation in genotypes (**Table S3**).

### Identification of QTL for PEL by GWAS

**Figure 3:**
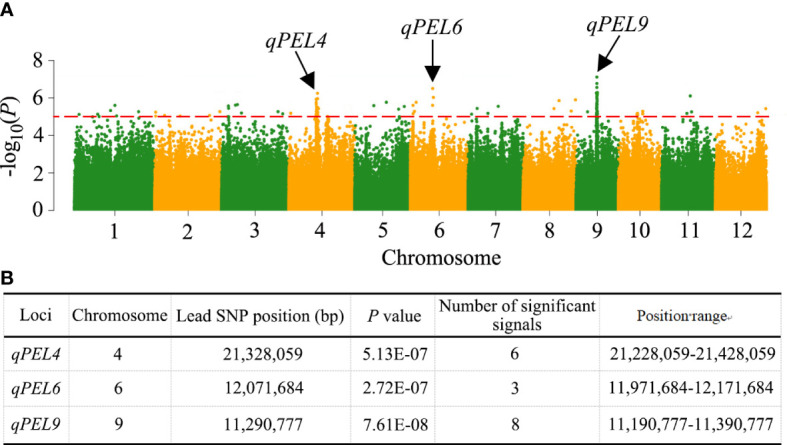
QTLs identified by GWAS in rice. **(A)** Manhattan plots for the whole population of 353 rice accessions. Negative log10 transformed P-values are plotted on the vertical axis, and dots above the red dashed line show the significant SNPs in the QTL region. The QTLs identified are shown by the black arrows. **(B)** Information about the identified QTLs.

We performed a GWAS between the PEL trait and SNPs (MAF >0.05) in the 353 rice accessions to investigate the possible genetic architecture of natural variation in PEL. In this population, 25 significantly associated SNP loci for PEL were detected in the 11 LD regions, which were located on chromosomes 2, 3, 4, 5, 6, 8, 9, and 11 (**Table 1**, **Figure 3A**). In addition, these 25 SNP loci were repeatedly detected in at least four environments, indicating that these association loci were stable (**Table S4**, **Figure S1**). The *R^2^* ranged from 3.50% to 8.44% (**Table 1**). When more than three significant SNP loci exceeded the threshold value (1 × 10^−5^) within a 200-kb interval, we considered the region a QTL based on the leading SNP. Three QTLs associated with PEL, *qPEL4*, *qPEL6*, and *qPEL9*, were identified (**Figure 3B**). Among them, the QTL*qPEL9* had the largest number (eight) of significant SNPs, suggesting that it is a major QTL for PEL. Next, we will focus on *qPEL9* for further analysis.

**Table 1:** The summary of SNPs significantly associated with PEL.

Chromosome	SNP site	Local LD	Allele	Range −Log_10_(*P*)	Range *R^2^*/%	Environment
2	317,322	317,322–317,322	G/T	5.04–5.34	3.50–4.10	E1, E3–E6
3	3,828,054	3,595,410–4,074,621	A/G	5.95–6.45	5.32–6.32	E1–E6
3	7,508,852	7,508,852–7,508,852	C/A	5.05–6.39	3.52–4.20	E1–E6
4	21,328,021	21,274,894–21,412,076	T/C	5.52–6.31	4.46–6.04	E1, E2, E4–E6
4	21,328,059	21,274,894–21,412,076	G/A	6.08–6.70	5.58–6.82	E1–E6
4	21,328,063	21,274,894–21,412,076	A/C	5.50–5.96	4.42–5.34	E1, E3–E6
4	21,328,066	21,274,894–21,412,076	A/T	5.61–6.47	4.64–6.36	E1–E4
4	21,328,085	21,274,894–21,412,076	G/A	5.56–5.88	4.54–5.18	E1–E6
4	21,328,088	21,274,894–21,412,076	G/T	5.78–6.48	4.98–6.38	E1–E3, E6
5	10,237,512	10,237,512–10,237,512	A/C	5.33–5.97	4.08–5.36	E1–E6
5	25,480,295	25,468,154–25,530,106	T/C	5.16–6.08	3.74–5.58	E1–E6
6	3,147,825	3,147,825–3,147,825	G/T	5.05–6.09	3.52–5.60	E1–E6
6	12,071,680	12,055,385–12,102,348	C/T	5.04–5.92	3.50–5.26	E1–E6
6	12,071,682	12,055,385–12,102,348	G/T	5.12–6.42	3.66–6.26	E2–E6
6	12,071,684	12,055,385–12,102,348	C/T	6.47–7.05	6.36–7.52	E1–E6
8	28,206,909	28,206,909–28,206,909	A/C	5.07–5.55	3.56–4.52	E1, E3–E6
9	11,289,927	11,239,027–11,329,539	G/A	5.51–5.95	4.44–5.32	E1–E6
9	11,290,285	11,239,027–11,329,539	T/C	5.80–6.97	5.02–7.36	E1, E3, E4, E6
9	11,290,484	11,239,027–11,329,539	T/A	5.56–6.85	4.54–7.12	E1–E6
9	11,290,697	11,239,027–11,329,539	G/A	5.38–6.79	4.18–7.00	E2, E3, E5, E6
9	11,290,715	11,239,027–11,329,539	G/A	6.36–6.68	6.14–6.78	E1–E6
9	11,290,777	11,239,027–11,329,539	T/G	6.96–7.51	7.34–8.44	E1–E6
9	11,291,418	11,239,027–11,329,539	A/G	5.51–6.77	4.44–6.96	E1, E2, E4–E6
9	11,291,449	11,239,027–11,329,539	C/T	6.59–7.07	6.60–7.56	E1–E6
11	15,537,689	15,537,689–15,537,689	C/A	6.22–6.70	5.86–6.82	E1–E6

LD, linkage disequilibrium; PEL, panicle elongation length. The value of −log_10_(P) indicates the significance levels and R^2^ indicates the percentage of phenotypic variation explained by each SNP. Environments: E1, environment 1, 2019 in Nanjing; E2, environment 2, 2020 in Nanjing; E3, environment 3, 2021 in Nanjing; E4, environment 4, 2019 in Hefei; E5, environment 5, 2020 in Hefei; E6, environment 6, 2021 in Hefei.

### *PEL9* is a causal gene for a PEL QTL

**Figure 4:**
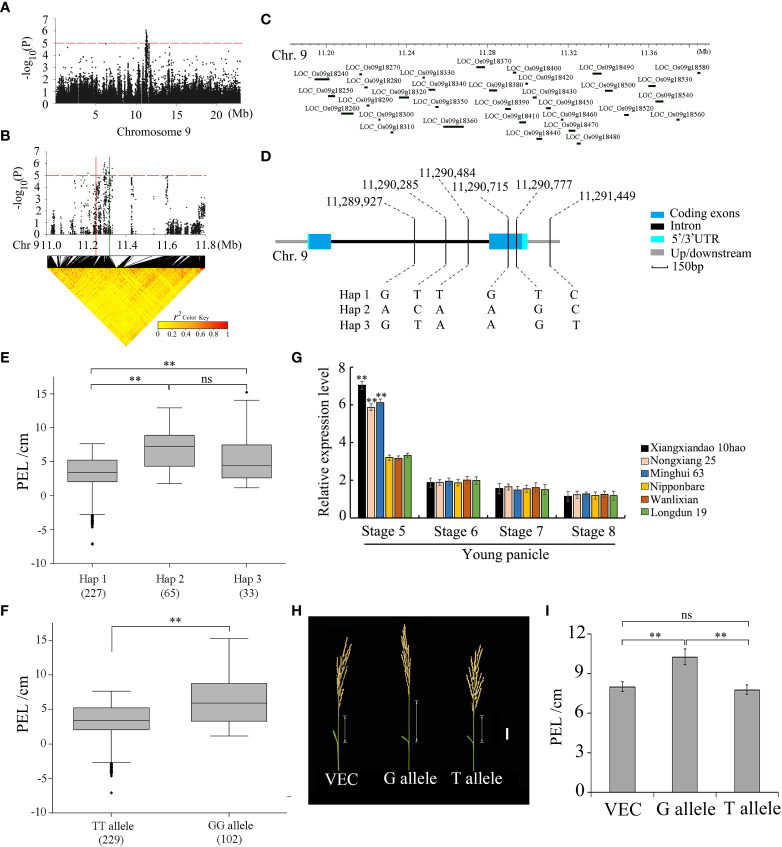
GWAS for PEL and identification of the causal gene *PEL9* (*LOC_Os09g18390*). **(A)** The Manhattan plot of chromosome 9 for PEL. In the Manhattan plots of chromosome 9, horizontal dashed lines indicate the significance threshold (−log10P = 5.0). **(B)** Local Manhattan plot (top) and LD heatmap (bottom). The arrow indicates the position of the nucleotide variation in *LOC_Os09g18390*. The candidate region lies between the solid red lines. **(C)** Identification of candidate genes in the region of *qPEL9.* **(D)** Haplotypes of *LOC_Os09g18390* associated with PEL in rice. **(E, F)** Boxplots of PEL in accessions containing the different haplotypes **(E)** and elite alleles **(F)**. The box edges represent the upper and lower quantiles. The median value in black was shown in the middle of the box. Vertical lines represent the data from the lowest quantile to the top quantile. Asterisks indicate individuals falling outside the range of the whiskers. Differences between alleles were statistically analyzed using a Student’s t-test (^**^ *P <*0.01). **(G)** Relative expression of *LOC_Os09g18390* in panicle below internode at development stages 5–8 of young panicle from the three accessions (Nipponbare, Nongxiang 25, and Longdun 19) with a long PEL and the three accessions (Xiangxiandao 10hao, Wanlixian, and Minghui 63) with a short PEL, determined by qRT-PCR (^**^ *P <*0.01, Student’s t-test). Data are presented as means ± s.e.; n = 3 independent biological replicates. **(H)** Images of panicles of transgenic plants transformed with the empty vector (VEC), the G allele, and the T allele. Scale bar, 3 cm. **(I)** PEL of transgenic plants. Data are presented as means ± s.e. (n = 20). PEL, panicle elongation length.

To search for candidate genes of *qPEL9*, the potential candidate region within 200 kb containing 100 kb upstream and 100 kb downstream of the significant leading SNP was analyzed (**Figures 4A, B**). In the 11.19–11.39 Mb candidate region, there were 32 candidate genes (**Figure 4C**). Combining with the LD block, we determined the LD block region as 11.24–11.33 Mb, which contained seven candidate genes (**Tables S5**, **S6**). Among them, five of the seven genes contained nonsynonymous SNPs (**Table S5**). Only the gene *LOC_Os09g18390* contained one nonsynonymous SNP significantly associated with the PEL. Hereafter, gene *LOC_Os09g18390* is referred to as *PEL9*.

The full length of *PEL9* is 2,030 bp, including two exons and one intron. Gene *PEL9* encodes a 172-amino acid protein. *PEL9* was classified into three haplotypes based on six SNPs in its cDNA, containing three SNPs in the intron region, two SNPs in the coding region, and one SNP in the downstream region (**Figure 4D**). The haplotype Hap 1 was associated with a shorter PEL, while haplotypes Hap 2 and Hap 3 were associated with a longer PEL (**Figure 4E**). For the two SNPs in the coding region, one SNP (11,290,715 bp) was synonymous, and one SNP (11,290,777 bp) was nonsynonymous (**Table S5**). The nonsynonymous SNP causes a base change from base G to base T at nt 510 bp in the coding sequence, which results in an amino acid change from serine (S) to isoleucine (I) at amino acid 164. The average PEL values of 229 accessions carrying the TT allele were 2.98 ± 3.23 cm, while those with the GG allele had a PEL of 6.51 ± 3.47 cm. There were highly significant differences between the TT allele and the GG allele at *P <*0.01 (**Figure 4F**).

We further performed quantitative RT-PCR analysis of the internode below young panicle at differentiation stages 5, 6, 7, and 8 of young panicle, sampled from three accessions (Nipponbare, Wanlixian, and Longdun 19) with longer PEL and three accessions (Xiangxiandao 10hao, Nongxiang 25, and Minghui 63) with shorter PEL, respectively. The qRT-PCR results showed that the expression level in the accessions with shorter PEL was much higher than that with longer PEL at stage 5 of young panicle, and no significant difference was found at stages 6, 7, and 8 of young panicle (**Figure 4G**). The accessions with longer PEL contained the allele *PEL9^GG^*, and the accessions with shorter PEL contained the allele *PEL9^TT^*. The expression of *PEL9^TT^* was the highest at stage 5 of the young panicle of the four stages investigated. However, the expression of *PEL9^GG^* did not peak in the internode below the young panicle among the four stages. These results suggested that decreased expression of *PEL9^GG^* might increase PEL.

Based on the results of GWAS, we found that no SNP loci located in the promoter region of *PEL9* were associated with PEL. Further, after searching the promoter functional elements website (http://bioinformatics.psb.ugent.be/webtools/plantcare/html/), we also found no SNP loci in the cis-element regulatory region. We speculated that the SNP loci in the CDS region caused the phenotypic variation between the accessions with the GG allele and those with the TT allele.

To validate the effect of the gene locus *PEL9* on PEL, we expressed the cDNA sequence from the alleles *PEL9* ^GG^ and *PEL9* ^TT^ under the control of a constitutive promoter in Nipponbare, which carries the allele *PEL9^GG^*, respectively. Compared with the empty vector control, plants overexpressing the allele *PEL9* ^GG^ showed a longer PEL, whereas no significant difference was observed for those overexpressing the allele *PEL9* ^TT^ (**Figures 4H, I**). These results indicate that *PEL9* is a causal gene for *qPEL9* on chromosome 9.

### Yield of the F_1_ hybrid seeds harvested from the male sterile line with the 7001S^*PEL9GG*^ allele was significantly higher than that with the 7001S^*PEL9TT*^ allele

**Figure 5:**
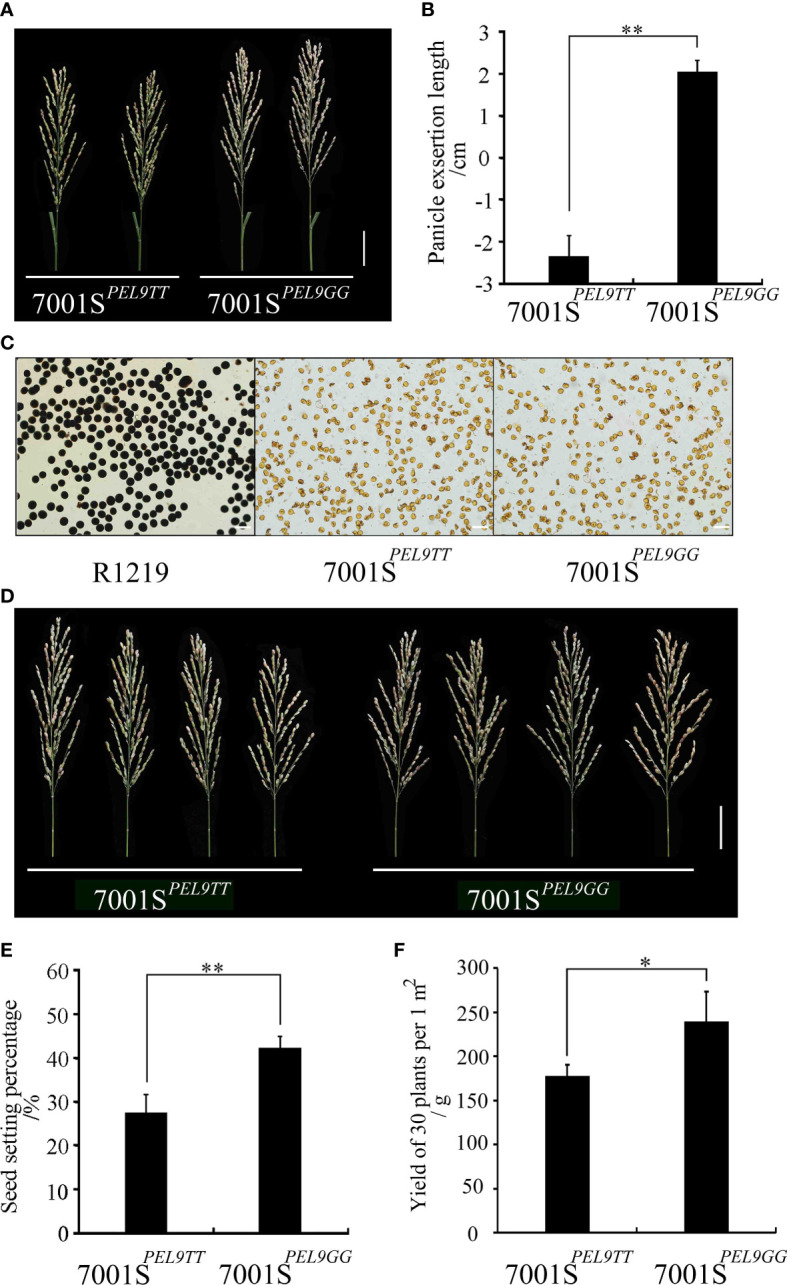
Hybrid rice seed production evaluation of *PEL9* gene. **(A)** Panicle morphology of 7001S*^PEL9TT^* and 7001S*^PEL9GG^*. Scale bar, 5 cm. **(B)** Comparison of the PELs of 7001S*^PEL9TT^* and 7001S*^PEL9GG^*. Data represent means ± SD (n = 35 independent plants), ^**^ *P <*0.01, Student’s *t*-test. **(C)** Pollen viability test. Fertile pollen grains of R1219 could be stained by 1% I_2_-KI, while abortive pollen grains of 7001S*^PEL9TT^* and 7001S*^PEL9GG^* could not be stained. Scale bar, 100 μm. **(D)** F_1_ panicle morphology of 7001S*^PEL9TT^* × R1219 and 7001S*^PEL9GG^* × R1219. Scale bar, 5 cm. **(E)** Comparison of the seed setting percentages of 7001S*^PEL9TT^* and 7001S*^PEL9GG^* after artificial supplementary pollination. Data represent means ± SD (n = 30 independent plants), ^**^ *P <*0.01, Student’s *t*-test. **(F)** Comparison of the yield of 30 plants per 1 m^2^ of 7001S*^PEL9TT^* and 7001S*^PEL9GG^* after artificial supplementary pollination. Data represent means ± SD (n = 30 independent plants), ^**^ *P <*0.01, Student’s *t*-test.

The PEL of 7001S*^PEL9GG^* was significantly longer than that of 7001S*^PEL9TT^* (**Figures 5A, B**). To further evaluate the potential of the *PEL9* allele in hybrid rice seed production, we performed a field experiment using two combinations, 7001S*^PEL9TT^* × R1219 and 7001S*^PEL9GG^* × R1219. The pollen of plants in 7001S*^PEL9GG^* and 7001S*^PEL9TT^* could not be stained by a 1% solution of I_2_-KI and was completely sterile (**Figure 5C**). So, F_1_ seeds were obtained from the plants in 7001S*^PEL9GG^* and 7001S*^PEL9TT^*, respectively. The out-crossing seed setting rate of the 7001S*^PEL9GG^* × R1219 combination was 42.27%, which is significantly (*P <*0.01) higher than that of the 7001S*^PEL9TT^* × R1219 combination (27.46%) (**Figures 5D, E**). The weight of the F_1_ seeds harvested from the female parents in the 1 m^2^ area for the combination of 7001S*^PEL9GG^* × R1219 was 238.54 g, which is significantly (*P <*0.05) higher than that of the combination of 7001S*^PEL9TT^* × R1219 (176.81 g) in the same area of land (**Figure 5F**). These results indicate that the *PEL9* ^GG^ allele could significantly increase the yield of F_1_ hybrid seeds by enhancing the out-crossing rate of the male sterile lines.

### Regional distribution of *PEL9* alleles

**Figure 6:**
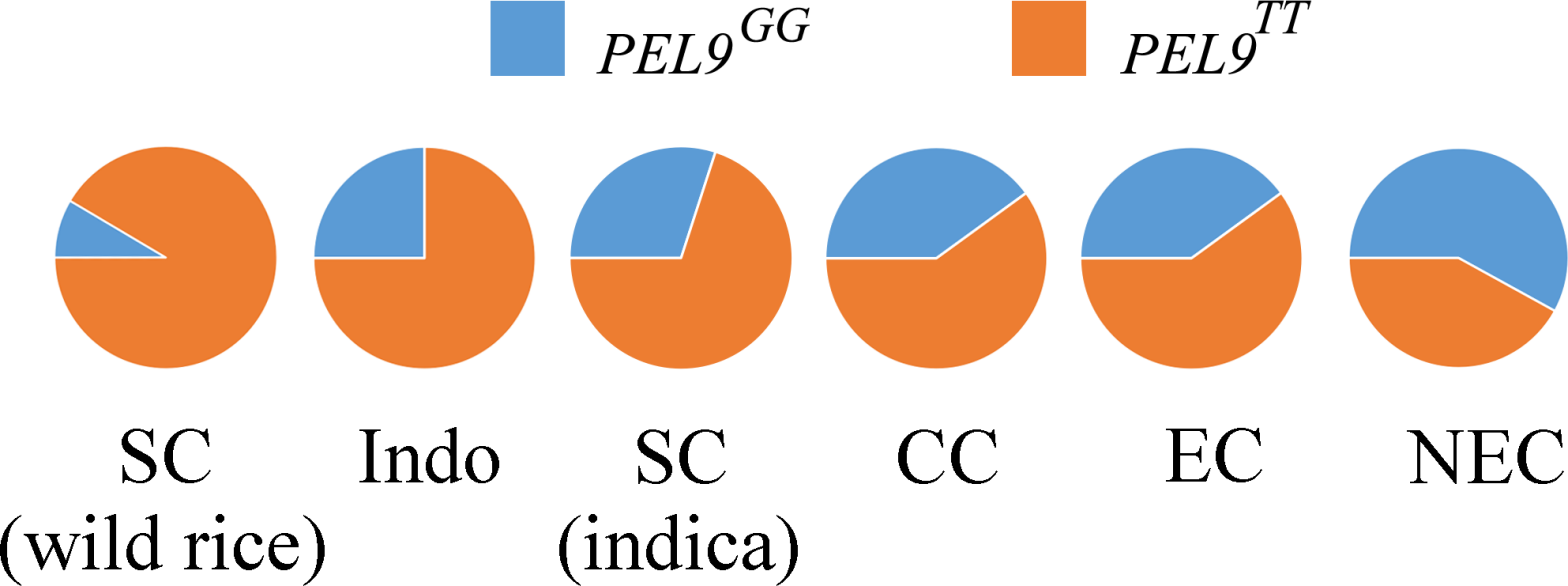
The gene allele frequency differences at the causal polymorphisms of *PEL9* in six geographic groups. The G allele indicates the type of reference allele. The T allele indicates the type of alternative allele. Indo, Indonesia; SC, southern China; CC, central China; EC, eastern China; NEC, northeastern China. The wild rice accessions were mainly from southern China. The accessions from Indo were mainly tropical *japonica* subspecies. The accessions from SC and CC were mainly *indica* subspecies. The accessions from EC and NEC were mainly temperate *japonica* subspecies.

To understand the geographical differentiation of *PEL9* alleles, we investigated the distribution of 353 *O. sativa* and 12 wild rice, including eight *O. rufipogon *and four *O. nivara* (Dang et al., 2020). In the 12 wild rice varieties, we found two alleles, *PEL9^GG^* and *PEL9^TT^*, and allele *PEL9^TT^* accounted for a larger proportion. For the 353 cultivated rice, a similar situation was observed for *PEL9*, in which the higher the latitude, the more significant the proportion of *PEL9^GG^* is (**Figure 6**). These results indicated that *PEL9^TT^* predominates at low latitudes and *PEL9^GG^* predominates at high latitudes.

To further confirm the allele frequency distribution of *PEL9*, we selected 446 wild rice (*Oryza rufipogon*) (http://server.ncgr.ac.cn/RiceHap3/GWAS.php) and 392 *O. sativa* (http://ricevarmap.ncpgr.cn/two_cultivars_compare/) (**Tables S7**-**S10**). The allele frequency distribution of *PEL9* in **Figure S2** was similar to that in **Figure 6** except tropical *japonica* (TRJ). This may have something to do with the source of the TRJ. The TRJ in this study were only from Indonesia, while the TRJ in the database were from many sources.

## Discussion

In this study, we investigated the phenotype data of PEL in 353 rice accessions across six environments and found that there existed a wide phenotypic variance. The coefficients of variation of PEL across six environments ranged from 89.83% to 91.91% (**Figure 2A**). Based on the results of a joint variance analysis, we know that the variations in PEL were caused mainly by the different genotypes and were rarely affected by the interactions between genotypes and environments (**Table S3**). These results mentioned above provide a reliable basis for the discovery of favorable alleles for the PEL trait.

The QTL regions detected in this study were based on the LD decay distance of each chromosome and significant peak SNP loci. This greatly increased the accuracy of candidate gene regions. In this study, we detected three stable QTLs, namely *qPEL4*, *qPEL6*, and *qPEL9* (**Figure 3**). Compared with the previously identified QTLs for PEL, two QTLs, *qPEL4* and *qPEL6*, were located at sites overlapping with the chromosome location of previously reported QTLs. The QTL *qPEL9* was found to be novel. The QTL *qPEL4* overlapped with the same region (20,171,917–22,349,484 bp) as the QTL *qPEN-4* reported by Hittalmani et al. (2002) and the QTL *qIN1–4* reported by Yamamoto et al. (2001). The QTL *qPEL6* was overlapped with the same region (8,115,201–17,152,315 bp) as the QTL *qLF-6* reported by Yang et al. (2011) and the QTL *qPEL6* reported by Dang et al. (2017). Compared with the previously cloned genes, no known genes were found in the QTL interval. We speculated that the reason was that the known genes were identified from mutants and the alleles from the known genes only existed in mutants and did not exist in the conventional material used in this study. Overall, the QTL *qPEL9* detected in this study is newly found, which provides a useful molecular basis for genetic improvement of PEL.

PEL is a component of the uppermost internode. The uppermost internode is a component of plant height (PH). Based on that, compared with the known genes controlling PH, such as *D1/RGA1* (Ashikari et al., 1999), *d11* (Zhu et al., 2015), *D2* (Fang et al., 2016), *d18/OsGA3ox2* (Tong et al., 2014; Hu et al., 2018)—controlling the shortening of the second internode length, *sd1/OsGA20ox2* (Spielmeyer et al., 2002), and *d35/OsKO2* (Itoh et al., 2004)—controlling the shortening of the uppermost internode length, and *d6/OSH15* (Fan et al., 2016)—controlling the shortening of two to four internode length and keeping of the uppermost internode length, we found that they were not in the same chromosome segments or in different segments of the same chromosome. These results indicated that the genetic basis of PEL was different from that of PH, which provided a theoretical basis for fine genetic improvement of PEL.

For one major QTL, *qPEL9*, we newly identified the potential causal gene *PEL9* (**Figure 4**). Gene *PEL9* encodes flavonol synthase (FLS)/flavanone 3-hydroxulase, which belongs to the 2-oxoglutarate-dependent dioxygenase and is a homolog of *Arabidopsis thaliana* FLS (AT5G08640, *AtFLS1*). *Arabidopsis AtFLS1* functions as a negative regulator of auxin transport *in vivo* and exhibits pleiotropic phenotypes including shoot length (Brown et al., 2001), whereas the function of its rice homolog was unknown. Combined with the results of qRT-PCR, the high expression of allele *PEL9^TT^* inhibited the auxin transport *in vivo* in rice, which resulted in short panicle elongation length, whereas the low expression of allele *PEL9^GG^* promoted the auxin transport *in vivo* in rice, which resulted in long panicle elongation length. We have demonstrated by the overexpression test that a base T-to-G nonsynonymous mutation at nt 510 in the coding sequence of *PEL9* caused the long PEL phenotype. Based on the field combination experiment, it was confirmed that the *PEL9* ^GG^ allele could significantly increase the yield of F_1_ hybrid seeds by enhancing the out-crossing rate of the sterile lines.

We also found two alleles of *PEL9^TT^* and *PEL9^GG^* in wild rice (**Figure 6** and **Figure S2**). And the proportion of the TT allele was greater than that of the GG allele. Except for TRJ, with an increase in latitude, the proportion of the TT allele decreased while the proportion of the GG allele increased. For TRJ, the proportion of the GG allele was greater than that of the TT allele. The allele frequency distribution in TRJ was different from that in other subgroups, and the dominant allele GG was the one that controlled the long PEL. We speculated that this may be a consequence of artificial selection. No matter in which high-latitude or low-latitude-high-altitude regions, conventional *japonica* rice is mainly planted. The *japonica* rice selected with panicle enclosure would affect rice quality and yield. Meanwhile, the long PEL facilitates pollination in F_1_ hybrid seed production. The accessions with the allele *PEL9* ^GG^ can be used to increase PEL in the maintainer lines (pollen parents used for multiplying the CMS lines) of hybrid japonica rice by crossing and marker-assisted selection breeding.

## Data availability statement

The datasets presented in this study can be found in online repositories. The names of the repository/repositories and accession number(s) can be found in the article/**Supplementary Material**.

## Author contributions

DH and JJ designed the experiments and managed the project. XD, CJ, MZ, XL, QX., CH, YL, YZ, and DW conducted field planting and phenotypic identification. XD, CJ, MZ, and XL performed the qRT-PCR and transformation analysis and the data analysis. XD wrote the manuscript draft, which was revised by DH and JJ. All authors listed have made a substantial, direct, and intellectual contribution to the work and approved it for publication.

## Supplementary material

Supplementary Figure 1 Manhattan plots and quantile-quantile plots depicting the results of genome-wide association study for the panicle elongation length trait using a mixed line model in the rice population composed of 353 accessions in each environment.

Supplementary Figure 2 The allele frequency at the causal polymorphisms of *PEL9*. The G allele indicates the type of reference allele. The T allele indicates the type of alternative allele. The sequence information of 446 wild rice and 392 Oryza sativa was downloaded from the website of and , respectively. WT, wild type; INDI, indica I; INDII, indica II; INDIII, indica III; TRJ, tropical *japonica*; TEJ, temperate *japonica*.
