# Supplementary figures and images for: A new allele PEL9 GG identified by genome-wide association study increases panicle elongation length in rice (Oryza sativa L.)
Source: Front Plant Sci. 2023 Feb 16;14:1136549. doi: 10.3389/fpls.2023.1136549 (PMC9978329; doi:10.3389/fpls.2023.1136549)

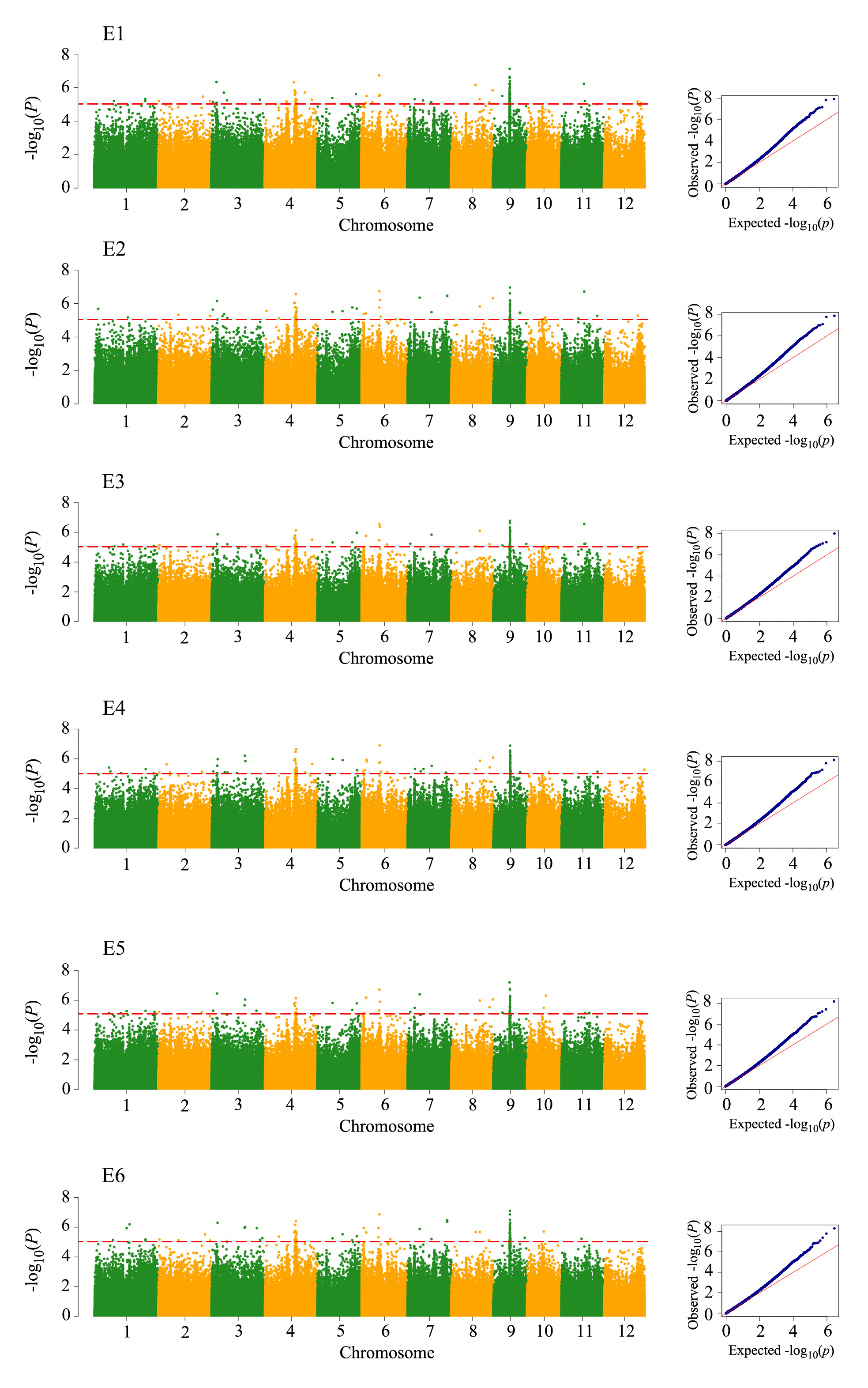

Supplement: Supplementary Figure 1 — Manhattan plots and quantile-quantile plots depicting the results of genome-wide association study for the panicle elongation length trait using a mixed line model in the rice population composed of 353 accessions in each environment. [file Image_1.tif]

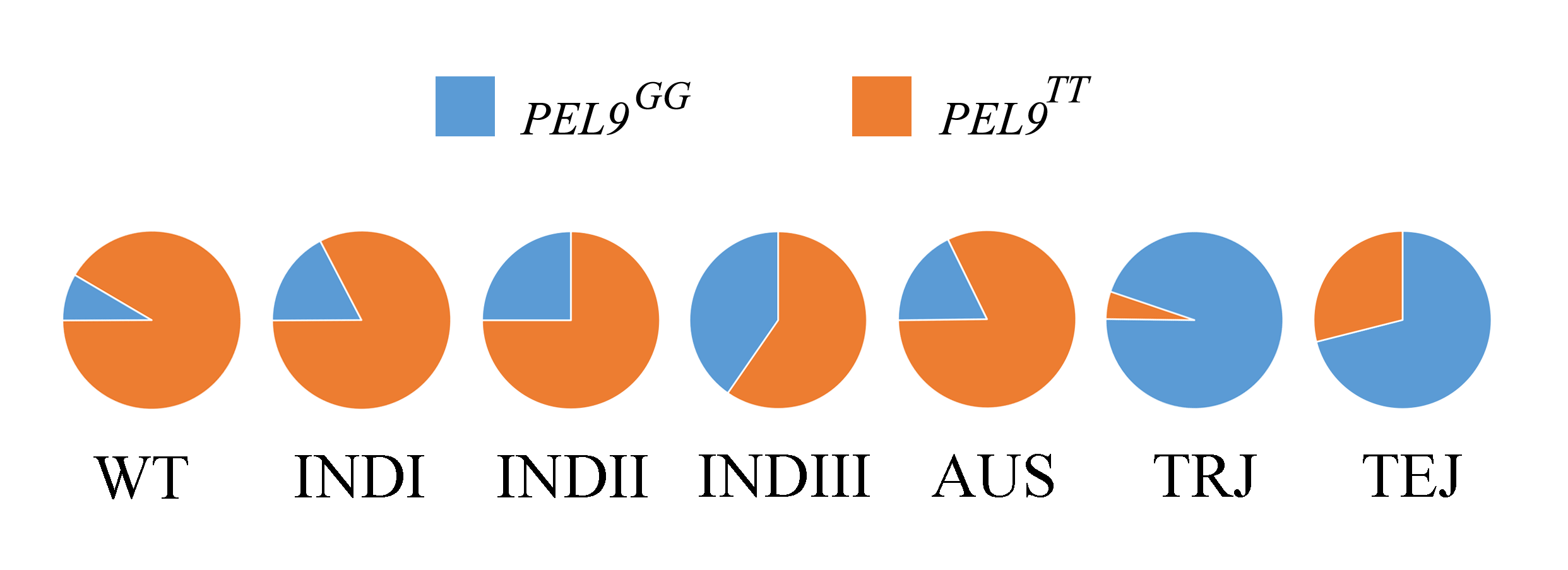

Supplement: Supplementary Figure 2 — The allele frequency at the causal polymorphisms of PEL9. The G allele indicates the type of reference allele. The T allele indicates the type of alternative allele. The sequence information of 446 wild rice and 392 Oryza sativa was downloaded from the website of and , respectively. WT, wild type; INDI, indica I; INDII, indica II; INDIII, indica III; TRJ, tropical japonica; TEJ, temperate japonica. [file Image_2.tif]
