# Supplementary material for: A new allele PEL9 GG identified by genome-wide association study increases panicle elongation length in rice (Oryza sativa L.)
Source: Front Plant Sci. 2023 Feb 16;14:1136549. doi: 10.3389/fpls.2023.1136549 (PMC9978329; doi:10.3389/fpls.2023.1136549)
Supplement: Supplementary file 4 [file Table_2.doc]

**Table S2. Primers used for this study.**

| Primer name | Forward primers (5’-3’) | Reverse primers (5’-3’) |
| --- | --- | --- |
| Primers for quantitative real-time RT-PCR: | | |
| RT1-18S | GAGATGGGTAGGGACGTGGAT | TGGTACGTCTCGTCCACCTT |
| RT2-LOC_Os09g18390 | GACGAGAGCTATGACCTGCC | GAAACCCCAGCTCCGACAAG |
| Primers for plasmid construction: | | |
| PEL9 | cagtGGTCTCacaacatggaaggctccaagctcatca | cagtGGTCTCatacattagacgagagatgaagaggac |
| Primers for genotype identification: | | |
|  | GCCAAGTAACCCACCCACAT | GACGAGAGATGAAGAGGACGA |
