# Supplementary material for: A new allele PEL9 GG identified by genome-wide association study increases panicle elongation length in rice (Oryza sativa L.)
Source: Front Plant Sci. 2023 Feb 16;14:1136549. doi: 10.3389/fpls.2023.1136549 (PMC9978329; doi:10.3389/fpls.2023.1136549)
Supplement: Supplementary file 5 [file Table_3.doc]

**Table S3.** The results of joint analysis of variance for the PEL trait.

| Traits | Source of variation | df | SS | MS | *F*-value | *F*_0.05_ | *F*_0.01_ |
| --- | --- | --- | --- | --- | --- | --- | --- |
| PEL/cm | Genotypes | 352 | 71155.2174 | 202.1455 | 151.22** | 1.18 | 1.26 |
|  | Environments | 5 | 122.3465 | 24.4693 | 0.20 | 4.39 | 8.75 |
|  | Genotype × Environment | 1760 | 2352.7515 | 1.3368 | 1.64** | 1.12 | 1.17 |

df, degrees of freedom; SS, sum of squares; MS, mean square.** Significant differences at *P* < 0.01. PEL, panicle elongation length.
