# Supplementary material for: A new allele PEL9 GG identified by genome-wide association study increases panicle elongation length in rice (Oryza sativa L.)
Source: Front Plant Sci. 2023 Feb 16;14:1136549. doi: 10.3389/fpls.2023.1136549 (PMC9978329; doi:10.3389/fpls.2023.1136549)
Supplement: Supplementary file 6 [file Table_4.docx]

**Table S4**. The distribution of the significant association SNP loci for PEL detected in more than 4 environments in this study.

| Trait | SNP site | The number of environments detected | E1 | | | E2 | | | E3 | | | E4 | | | E5 | | | E6 | | |
| --- | --- | --- | --- | --- | --- | --- | --- | --- | --- | --- | --- | --- | --- | --- | --- | --- | --- | --- | --- | --- |
|  |  |  | *P* value | R2% | FDR | *P* value | R2% | FDR | *P* value | R2% | FDR | *P* value | R2% | FDR | *P* value | R2% | FDR | *P* value | R2% | FDR |
| PEL | 317,322 | 5 | 7.51E-06 | 3.66 | 5.31E-06 |  |  |  | 9.20E-06 | 3.50 | 8.52E-06 | 8.53E-06 | 3.52 | 7.29E-06 | 4.58E-06 | 4.10 | 3.40E-06 | 6.68E-06 | 3.78 | 5.33E-06 |
|  | 3,828,054 | 6 | 4.61E-07 | 6.10 | 3.15E-07 | 5.08E-07 | 6.00 | 4.62E-07 | 1.11E-06 | 5.32 | 1.04E-06 | 1.00E-06 | 5.42 | 7.48E-07 | 5.94E-07 | 5.88 | 4.39E-07 | 3.56E-07 | 6.32 | 2.23E-07 |
|  | 7,508,852 | 6 | 4.07E-07 | 4.20 | 1.08E-06 | 6.48E-06 | 3.80 | 5.64E-06 | 6.70E-06 | 3.76 | 5.95E-06 | 8.60E-06 | 3.56 | 7.47E-06 | 8.65E-06 | 3.54 | 7.36E-06 | 8.85E-06 | 3.52 | 2.46E-07 |
|  | 21,328,021 | 5 | 5.73E-07 | 5.9 | 3.91E-07 | 4.85E-07 | 6.04 | 3.52E-07 |  |  |  | 1.75E-06 | 4.94 | 1.43E-06 | 2.99E-06 | 4.46 | 1.22E-06 | 1.28E-06 | 5.20 | 1.12E-07 |
|  | 21,328,059 | 6 | 4.87E-07 | 6.04 | 3.23E-07 | 2.00E-07 | 6.82 | 1.51E-07 | 8.38E-07 | 5.58 | 7.21E-07 | 2.13E-07 | 6.76 | 1.31E-07 | 5.22E-07 | 5.98 | 4.57E-07 | 3.62E-07 | 6.30 | 2.30E-07 |
|  | 21,328,063 | 5 | 2.98E-06 | 4.48 | 2.03E-06 |  |  |  | 3.13E-06 | 4.42 | 2.86E-06 | 2.82E-06 | 4.52 | 1.64E-06 | 2.99E-06 | 4.46 | 1.72E-06 | 1.10E-06 | 5.34 | 1.09E-07 |
|  | 21,328,066 | 4 | 2.17E-06 | 4.74 | 1.39E-06 | 1.27E-06 | 5.22 | 1.03E-06 | 2.47E-06 | 4.64 | 2.02E-06 | 3.38E-07 | 6.36 | 2.42E-07 |  |  |  |  |  |  |
|  | 21,328,085 | 6 | 2.73E-06 | 4.54 | 2.23E-06 | 1.95E-06 | 4.84 | 1.77E-06 | 2.00E-06 | 4.82 | 1.83E-06 | 1.83E-06 | 4.90 | 1.32E-06 | 1.33E-06 | 5.18 | 1.03E-06 | 2.33E-06 | 4.68 | 1.85E-06 |
|  | 21,328,088 | 4 | 1.28E-06 | 5.22 | 1.00E-06 | 1.66E-06 | 4.98 | 1.59E-06 | 1.32E-06 | 5.18 | 1.19E-06 |  |  |  |  |  |  | 3.31E-07 | 6.38 | 2.53E-07 |
|  | 10,237,512 | 6 | 3.41E-06 | 4.36 | 3.02E-06 | 2.95E-06 | 4.48 | 2.78E-06 | 3.92E-06 | 4.24 | 3.62E-06 | 1.07E-06 | 5.36 | 7.52E-07 | 1.28E-06 | 5.20 | 1.01E-06 | 4.73E-06 | 4.08 | 3.42E-06 |
|  | 25,480,295 | 6 | 1.94E-06 | 4.84 | 1.57E-06 | 2.22E-06 | 4.72 | 1.98E-06 | 8.28E-07 | 5.58 | 7.29E-07 | 6.90E-06 | 3.74 | 5.14E-06 | 1.34E-06 | 5.16 | 1.03E-06 | 3.02E-06 | 4.46 | 2.21E-06 |
|  | 3,147,825 | 6 | 3.28E-06 | 4.38 | 2.18E-06 | 8.85E-06 | 3.52 | 8.07E-06 | 1.91E-06 | 4.86 | 1.46E-06 | 1.27E-06 | 5.22 | 1.11E-06 | 8.10E-07 | 5.60 | 7.42E-07 | 2.50E-06 | 4.52 | 1.92E-06 |
|  | 12,071,680 | 6 | 2.78E-06 | 4.54 | 2.33E-06 | 2.51E-06 | 4.62 | 2.13E-06 | 3.03E-06 | 4.46 | 2.47E-06 | 9.20E-06 | 3.50 | 8.81E-06 | 1.21E-06 | 5.26 | 9.80E-07 | 3.41E-06 | 4.36 | 2.54E-06 |
|  | 12,071,682 | 5 |  |  |  | 5.38E-07 | 5.96 | 4.90E-07 | 3.84E-07 | 6.26 | 2.89E-07 | 1.57E-06 | 5.02 | 1.32E-06 | 7.51E-06 | 3.66 | 5.41E-06 | 8.60E-07 | 5.56 | 7.56E-07 |
|  | 12,071,684 | 6 | 2.56E-07 | 6.6 | 2.06E-07 | 2.47E-07 | 6.64 | 2.00E-07 | 2.67E-07 | 6.56 | 2.22E-07 | 3.41E-07 | 6.36 | 2.33E-07 | 2.65E-07 | 6.58 | 1.75E-07 | 8.85E-08 | 7.52 | 7.46E-08 |
|  | 28,206,909 | 5 | 7.25E-06 | 3.70 | 5.12E-06 |  |  |  | 5.96E-06 | 3.86 | 5.49E-06 | 2.85E-06 | 4.52 | 2.45E-06 | 2.95E-06 | 4.48 | 2.38E-06 | 8.60E-06 | 3.56 | 7.56E-06 |
|  | 11,289,927 | 6 | 1.12E-06 | 5.32 | 8.70E-07 | 3.03E-06 | 4.46 | 2.76E-06 | 3.07E-06 | 4.44 | 2.46E-06 | 1.92E-06 | 4.86 | 1.57E-06 | 2.17E-06 | 4.74 | 1.80E-06 | 1.13E-06 | 5.32 | 1.08E-07 |
|  | 11,290,285 | 4 | 1.58E-06 | 5.02 | 1.20E-06 |  |  |  | 1.82E-07 | 6.90 | 1.35E-07 | 2.13E-07 | 6.76 | 1.92E-07 |  |  |  | 1.07E-07 | 7.36 | 6.70E-08 |
|  | 11,290,484 | 6 | 2.73E-06 | 4.54 | 2.41E-06 | 2.04E-07 | 6.80 | 1.92E-07 | 3.18E-07 | 6.42 | 2.89E-07 | 1.42E-07 | 7.12 | 1.08E-07 | 2.61E-06 | 6.58 | 1.90E-07 | 2.70E-07 | 6.56 | 1.56E-07 |
|  | 11,290,697 | 4 |  |  |  | 3.04E-07 | 6.46 | 2.67E-07 | 4.18E-06 | 4.18 | 3.82E-06 |  |  |  | 1.61E-07 | 7.00 | 1.23E-07 | 3.70E-07 | 6.28 | 2.45E-07 |
|  | 11,290,715 | 6 | 2.58E-07 | 6.60 | 1.70E-07 | 2.92E-07 | 6.48 | 2.65E-07 | 2.82E-07 | 6.52 | 1.62E-07 | 3.13E-07 | 6.42 | 2.77E-07 | 4.36E-07 | 6.14 | 1.42E-07 | 2.07E-07 | 6.78 | 1.01E-07 |
|  | 11,290,777 | 6 | 3.15E-08 | 8.42 | 1.62E-08 | 7.23E-08 | 7.70 | 6.22E-08 | 1.10E-07 | 7.34 | 9.20E-08 | 8.92E-08 | 7.52 | 7.45E-08 | 3.11E-08 | 8.44 | 2.60E-08 | 3.15E-08 | 8.42 | 2.20E-08 |
|  | 11,291,418 | 5 | 1.40E-06 | 5.12 | 1.08E-06 | 3.06E-06 | 4.44 | 2.76E-06 |  |  |  | 1.24E-06 | 5.24 | 1.18E-06 | 2.51E-06 | 4.62 | 1.65E-06 | 1.70E-07 | 6.96 | 1.50E-07 |
|  | 11,291,449 | 6 | 9.38E-08 | 7.48 | 7.01E-08 | 1.57E-07 | 7.02 | 1.11E-07 | 2.55E-07 | 6.60 | 1.72E-07 | 2.17E-07 | 6.74 | 2.01E-07 | 1.24E-07 | 7.24 | 1.05E-07 | 8.45E-08 | 7.56 | 6.64E-08 |
|  | 15,537,689 | 6 | 5.96E-07 | 5.86 | 4.87E-07 | 2.67E-07 | 6.56 | 2.11E-07 | 4.56E-07 | 6.10 | 3.84E-07 | 2.97E-07 | 6.48 | 2.47E-07 | 3.84E-07 | 6.26 | 2.72E-07 | 2.01E-07 | 6.82 | 1.14E-07 |

PEL, panicle elongation length; E1, environment 1; E2, environment 2; E3, environment 3; E4, environment 4; E5, environment 5; E6, environment 6.
