# Supplementary material for: A new allele PEL9 GG identified by genome-wide association study increases panicle elongation length in rice (Oryza sativa L.)
Source: Front Plant Sci. 2023 Feb 16;14:1136549. doi: 10.3389/fpls.2023.1136549 (PMC9978329; doi:10.3389/fpls.2023.1136549)
Supplement: Supplementary file 8 [file Table_6.doc]

**Table S6**. Candidate gene annotation in the LD region 11.24-11.33Mb associated with panicle elongation length.

| Number | MSU ID | Position | Annotation |
| --- | --- | --- | --- |
| 1 | LOC_Os09g18320 | 11,236,537-11,240,954 | bystin, expressed protein |
| 2 | LOC_Os09g18360 | 11,258,656-11,268,043 | expressed protein |
| 3 | LOC_Os09g18390 | 11,288,812-11,290,841 | flavonol synthase/flavanone 3-hydroxylase |
| 4 | LOC_Os09g18450 | 11,309,063-11,310,776 | flavonol synthase/flavanone 3-hydroxylase |
| 5 | LOC_Os09g18470 | 11,320,295-11,322,989 | oxidoreductase, 2OG-Fe oxygenase family protein |
| 6 | LOC_Os09g18480 | 11,324,824-11,326,092 | retrotransposon protein |
| 7 | LOC_Os09g18490 | 11,332,585-11,336,337 | transposon protein |
