# Supplementary material for: A new allele PEL9 GG identified by genome-wide association study increases panicle elongation length in rice (Oryza sativa L.)
Source: Front Plant Sci. 2023 Feb 16;14:1136549. doi: 10.3389/fpls.2023.1136549 (PMC9978329; doi:10.3389/fpls.2023.1136549)
Supplement: Supplementary file 9 [file Table_7.doc]

**Table S7**. Base information of 446 *Oryza rufipogon* reported by Huang et al. (2012) (https://www.ebi.ac.uk/ena/browser/view/PRJEB2829)

| Code | ID name | Original area | Latitude | Longitude | Code | ID name | Original area | Latitude | Longitude |
| --- | --- | --- | --- | --- | --- | --- | --- | --- | --- |
| 1 | W0101 | India | 20.48 | 85.85 | 224 | W1859 | Thailand | 17.52 | 100.12 |
| 2 | W0102 | India | 20.48 | 85.85 | 225 | W1862 | Thailand | 16.82 | 100.34 |
| 3 | W0103 | India | 20.48 | 85.85 | 226 | W1865 | Thailand | 14.56 | 100.98 |
| 4 | W0106 | India | 20.46 | 85.88 | 227 | W1866 | Thailand | 14.57 | 100.99 |
| 5 | W0107 | India | 20.95 | 85.1 | 228 | W1870 | Thailand | 15.23 | 102.5 |
| 6 | W0108 | India | 20.46 | 85.88 | 229 | W1873 | Thailand | 16.35 | 102.86 |
| 7 | W0120 | India | 20.46 | 85.88 | 230 | W1879 | Thailand | 17.03 | 102.91 |
| 8 | W0121 | India | 20.46 | 85.88 | 231 | W1880 | Thailand | 17.03 | 102.91 |
| 9 | W0123 | India | 20.46 | 85.93 | 232 | W1881 | Thailand | 17.03 | 102.91 |
| 10 | W0124 | India | 21 | 85.1 | 233 | W1882 | Thailand | 17.03 | 102.91 |
| 11 | W0125 | India | 21 | 85.1 | 234 | W1884 | Thailand | 17.03 | 102.91 |
| 12 | W0126 | India | 21 | 85.1 | 235 | W1890 | Thailand | 17.85 | 102.75 |
| 13 | W0128 | India | 21 | 85.1 | 236 | W1891 | Thailand | 17.85 | 102.75 |
| 14 | W0130 | India | 21 | 85.1 | 237 | W1893 | Thailand | 17.35 | 102.89 |
| 15 | W0132 | India | 17.05 | 82.18 | 238 | W1895 | Thailand | 17.25 | 104.18 |
| 16 | W0133 | India | 17.05 | 82.18 | 239 | W1896 | Thailand | 17.25 | 104.18 |
| 17 | W0134 | India | 16.93 | 81.88 | 240 | W1912 | Thailand | 15.05 | 104.1 |
| 18 | W0135 | India | 16.93 | 81.88 | 241 | W1914 | Thailand | 14.95 | 103.75 |
| 19 | W0136 | India | 16.91 | 81.82 | 242 | W1916 | Thailand | 14.92 | 103.51 |
| 20 | W0137 | India | 16.91 | 81.82 | 243 | W1919 | Thailand | 14.73 | 102.19 |
| 21 | W0138 | India | 10.51 | 76.65 | 244 | W1921 | Thailand | 14.44 | 100.9 |
| 22 | W0141 | India | 10.37 | 76.37 | 245 | W1925 | Thailand | 15.55 | 100.12 |
| 23 | W0143 | Sri Lanka | 7.21 | 80.2 | 246 | W1927 | Thailand | 15.46 | 100.13 |
| 24 | W0144 | Sri Lanka | 7.07 | 80.3 | 247 | W1928 | Thailand | 15.29 | 100.18 |
| 25 | W0145 | Thailand | 13.72 | 100.48 | 248 | W1935 | Thailand | 6.89 | 100.53 |
| 26 | W0147 | India | 20.48 | 85.85 | 249 | W1939 | Thailand | 8.54 | 99.73 |
| 27 | W0148 | India | 20.48 | 85.85 | 250 | W1940 | Thailand | 14.92 | 103.51 |
| 28 | W0149 | India | 23.48 | 81.1 | 251 | W1943 | China | NA | NA |
| 29 | W0151 | India | 23.48 | 81.1 | 252 | W1945 | China | NA | NA |
| 30 | W0152 | India | 22.9 | 88.25 | 253 | W1952 | China | NA | NA |
| 31 | W0153 | India | 22.4 | 88.66 | 254 | W1957 | China | NA | NA |
| 32 | W0157 | India | 26.59 | 94.19 | 255 | W1958 | China | NA | NA |
| 33 | W0163 | Thailand | 18.81 | 98.66 | 256 | W1959 | China | NA | NA |
| 34 | W0164 | Thailand | 18.81 | 98.66 | 257 | W1963 | China | NA | NA |
| 35 | W0165 | Thailand | 18.81 | 98.66 | 258 | W1970 | Indonesia | -6.4 | 106.82 |
| 36 | W0166 | Thailand | 18.81 | 98.66 | 259 | W1971 | Indonesia | -6.4 | 106.82 |
| 37 | W0168 | Thailand | 15.22 | 102.5 | 260 | W1972 | Indonesia | -6.4 | 106.82 |
| 38 | W0169 | Thailand | 15.87 | 100.99 | 261 | W1973 | Indonesia | -6.4 | 106.82 |
| 39 | W0170 | Thailand | 15.87 | 100.99 | 262 | W1974 | Indonesia | -2.99 | 104.76 |
| 40 | W0171 | Thailand | 15.87 | 100.99 | 263 | W1975 | Indonesia | -2.99 | 104.76 |
| 41 | W0172 | Thailand | 15.87 | 100.99 | 264 | W1976 | Indonesia | -6.4 | 106.82 |
| 42 | W0173 | Thailand | 15.87 | 100.99 | 265 | W1977 | Indonesia | -6.4 | 106.82 |
| 43 | W0174 | Thailand | 18.08 | 103.27 | 266 | W1978 | Indonesia | -6.4 | 106.82 |
| 44 | W0175 | Thailand | 18.08 | 103.27 | 267 | W1979 | Indonesia | -6.4 | 106.82 |
| 45 | W0176 | Thailand | 18.08 | 103.27 | 268 | W1981 | Indonesia | -2.99 | 104.76 |
| 46 | W0178 | Thailand | 14.33 | 100.53 | 269 | W1983 | India | 18.5 | 73.15 |
| 47 | W0179 | Thailand | 17.42 | 102.78 | 270 | W1989 | India | 20.3 | 73 |
| 48 | W0180 | Thailand | 18.77 | 99.97 | 271 | W1990 | India | 23.1 | 72.45 |
| 49 | W0234 | Thailand | 18.08 | 103.27 | 272 | W1991 | India | 22.3 | 71.05 |
| 50 | W0549 | India | 13 | 77.6 | 273 | W1993 | India | 24.6 | 72.8 |
| 51 | W0573 | Malaya | 3.1 | 101.5 | 274 | W1995 | India | 22.42 | 73 |
| 52 | W0574 | Malaya | 3.1 | 101.5 | 275 | W1998 | India | 22.2 | 73.2 |
| 53 | W0576 | Malaya | 5.8 | 102.38 | 276 | W2003 | India | 15.3 | 73.5 |
| 54 | W0587 | Malaya | 5.7 | 102.53 | 277 | W2005 | India | 15.35 | 74.2 |
| 55 | W0589 | Malaya | 5.7 | 102.53 | 278 | W2007 | India | 16 | 74.3 |
| 56 | W0590 | Malaya | 5.7 | 102.53 | 279 | W2008 | India | 16.2 | 74.2 |
| 57 | W0593 | Malaya | 3.14 | 101.69 | 280 | W2010 | India | 19 | 73.06 |
| 58 | W0594 | Malaya | 3.14 | 101.69 | 281 | W2012 | India | 19.8 | 72.55 |
| 59 | W0596 | Malaya | 3.14 | 101.69 | 282 | W2014 | India | 20.18 | 72.55 |
| 60 | W0600 | Malaya | 5.14 | 102.81 | 283 | W2017 | Indonesia | -7.6 | 110.7 |
| 61 | W0605 | Malaya | 5.8 | 102.39 | 284 | W2021 | Indonesia | 3.29 | 117 |
| 62 | W0606 | Malaya | 6 | 102.25 | 285 | W2022 | Indonesia | 3.29 | 117 |
| 63 | W0610 | Burma | 16.8 | 96.15 | 286 | W2024 | Indonesia | 3.29 | 117 |
| 64 | W0621 | Burma | 17.33 | 96.5 | 287 | W2025 | Indonesia | 3.29 | 117 |
| 65 | W0623 | Burma | 17.55 | 96.62 | 288 | W2030 | Indonesia | -3 | 105 |
| 66 | W0624 | Burma | 19.77 | 96.11 | 289 | W2036 | Burma | 17.33 | 96.5 |
| 67 | W0625 | Burma | 19.77 | 96.11 | 290 | W2050 | Bangladesh | 23.71 | 90.41 |
| 68 | W0626 | Burma | 19.77 | 96.11 | 291 | W2051 | Bangladesh | 23.71 | 90.41 |
| 69 | W0627 | Burma | 19.77 | 96.11 | 292 | W2052 | Bangladesh | 23.71 | 90.41 |
| 70 | W0628 | Burma | 20.4 | 92.85 | 293 | W2053 | Bangladesh | 23.71 | 90.41 |
| 71 | W0629 | Burma | 20.4 | 92.85 | 294 | W2055 | Bangladesh | 24.48 | 91.78 |
| 72 | W0630 | Burma | 20.46 | 94.56 | 295 | W2056 | Bangladesh | 24.48 | 91.78 |
| 73 | W0631 | Burma | 18.82 | 95.22 | 296 | W2057 | Bangladesh | 24.48 | 91.78 |
| 74 | W0632 | Burma | 18.82 | 95.22 | 297 | W2060 | Bangladesh | 24.25 | 89.92 |
| 75 | W0633 | Burma | 18.82 | 95.22 | 298 | W2061 | Bangladesh | 24.25 | 89.92 |
| 76 | W0634 | Burma | 25.38 | 97.39 | 299 | W2063 | Bangladesh | 22.82 | 89.55 |
| 77 | W0635 | Burma | 19.77 | 96.11 | 300 | W2064 | Bangladesh | 22.82 | 89.55 |
| 78 | W0637 | Burma | 14.08 | 98.2 | 301 | W2066 | Nepal | 28.6 | 81.6 |
| 79 | W0638 | Burma | 14.08 | 98.2 | 302 | W2078 | Australia | -14.3 | 132.4 |
| 80 | W0639 | Burma | 19.77 | 96.11 | 303 | W2099 | Australia | -13.07 | 142.07 |
| 81 | W1080 | India | 27 | 88.4 | 304 | W2108 | Australia | -13.07 | 142.07 |
| 82 | W1082 | India | 27 | 88.4 | 305 | W2193 | India | 24.83 | 93.93 |
| 83 | W1083 | India | 27 | 88.4 | 306 | W2197 | Indonesia | 3.29 | 117 |
| 84 | W1084 | India | 27 | 88.4 | 307 | W2198 | China | NA | NA |
| 85 | W1086 | India | 27 | 88.4 | 308 | W2263 | Cambodia | 11.33 | 104.5 |
| 86 | W1087 | India | 26.15 | 91.74 | 309 | W2264 | Vietnam | 10.2 | 105.47 |
| 87 | W1090 | India | 26.15 | 91.74 | 310 | W2265 | Laos | 14.5 | 105.49 |
| 88 | W1092 | India | 26.15 | 91.74 | 311 | W2266 | Laos | 18.01 | 102.39 |
| 89 | W1093 | India | 26.15 | 91.74 | 312 | W2267 | Laos | 18.14 | 102.42 |
| 90 | W1096 | India | 26.2 | 92.94 | 313 | W2268 | Thailand | 14.35 | 101 |
| 91 | W1102 | India | 26.2 | 92.94 | 314 | W2269 | Thailand | 17.42 | 102.46 |
| 92 | W1105 | India | 26.82 | 94.17 | 315 | W2271 | Thailand | 16.57 | 102.55 |
| 93 | W1107 | India | 26.82 | 94.17 | 316 | W2272 | Thailand | 16.21 | 102.48 |
| 94 | W1111 | India | 26.82 | 94.17 | 317 | W2275 | Thailand | 16.09 | 100.37 |
| 95 | W1112 | India | 26.82 | 94.17 | 318 | W2276 | Thailand | 16.49 | 100.21 |
| 96 | W1114 | India | 26.82 | 94.17 | 319 | W2277 | Thailand | 15.13 | 100.12 |
| 97 | W1117 | India | 26.82 | 94.17 | 320 | W2278 | Thailand | 14.3 | 100.31 |
| 98 | W1119 | India | 26.82 | 94.17 | 321 | W2282 | Thailand | 16.49 | 99.47 |
| 99 | W1121 | India | 26.82 | 94.17 | 322 | W2283 | Thailand | 16.49 | 99.47 |
| 100 | W1122 | India | 26.82 | 94.17 | 323 | W2284 | Thailand | 16.49 | 99.47 |
| 101 | W1124 | India | 26.59 | 94.2 | 324 | W2288 | Cambodia | 11.04 | 106.09 |
| 102 | W1126 | India | 24.86 | 92.36 | 325 | W2296 | Cambodia | 11.32 | 104.5 |
| 103 | W1142 | India | 20.46 | 85.93 | 326 | W2298 | Laos | 15.09 | 105.46 |
| 104 | W1143 | India | 20.46 | 85.93 | 327 | W2299 | Laos | 15.09 | 105.46 |
| 105 | W1161 | Sri Lanka | 6.55 | 80.1 | 328 | W2301 | Laos | 15.12 | 105.43 |
| 106 | W1214 | Philippines | 7.86 | 124.86 | 329 | W2302 | Laos | 15.12 | 105.43 |
| 107 | W1230 | Dutch New Guinea | -4.63 | 138.93 | 330 | W2303 | Laos | 15.06 | 105.49 |
| 108 | W1236 | Australian New Guinea | -5.31 | 141.61 | 331 | W2304 | Laos | 15.03 | 105.54 |
| 109 | W1238 | Neth. New Guinea | -4.63 | 138.93 | 332 | W2305 | Laos | 14.5 | 105.5 |
| 110 | W1244 | Nepal | 27.7 | 85.32 | 333 | W2306 | Laos | 14.5 | 105.5 |
| 111 | W1292 | Indonesia | 3.29 | 117 | 334 | W2307 | Laos | 14.52 | 105.52 |
| 112 | W1294 | Philippines | 7.86 | 124.86 | 335 | W2308 | Laos | 17.57 | 102.38 |
| 113 | W1295 | Cambodia | 12.82 | 102.67 | 336 | W2310 | Laos | 17.51 | 102.36 |
| 114 | W1532 | India | 20.46 | 85.88 | 337 | W2311 | Laos | 17.52 | 102.36 |
| 115 | W1533 | India | 20.46 | 85.88 | 338 | W2316 | Vietnam | 10.39 | 107.02 |
| 116 | W1534 | India | 28.64 | 77.23 | 339 | W2318 | Vietnam | 10.24 | 106.06 |
| 117 | W1536 | Sri Lanka | 8.03 | 79.84 | 340 | W2319 | Vietnam | 10.33 | 106.25 |
| 118 | W1542 | Malaya | 3.14 | 101.69 | 341 | W2320 | Vietnam | 10.42 | 105.36 |
| 119 | W1546 | Thailand | 14.5 | 100.89 | 342 | W2321 | Vietnam | 10.44 | 105.37 |
| 120 | W1547 | Thailand | 14.5 | 100.89 | 343 | W2322 | Vietnam | 10.45 | 105.32 |
| 121 | W1550 | Thailand | 18.8 | 98.66 | 344 | W2327 | Vietnam | 10 | 105.45 |
| 122 | W1551 | Thailand | 14.5 | 100.89 | 345 | W2331 | Vietnam | 21.03 | 105.85 |
| 123 | W1552 | Thailand | 14.33 | 100.52 | 346 | W2332 | Vietnam | 21.03 | 105.85 |
| 124 | W1553 | Thailand | 14.6 | 103.1 | 347 | W3000 | China | 21.38 | 110.25 |
| 125 | W1554 | Thailand | 15.09 | 99.99 | 348 | W3001 | China | 21.38 | 110.25 |
| 126 | W1555 | Thailand | 15 | 100 | 349 | W3002 | China | 22.19 | 112.31 |
| 127 | W1556 | Thailand | 14.54 | 99.99 | 350 | W3003 | China | 22.19 | 112.31 |
| 128 | W1557 | Thailand | 14.54 | 99.99 | 351 | W3004 | China | 22.25 | 112.79 |
| 129 | W1558 | Thailand | 15.2 | 104.9 | 352 | W3005 | China | 22.25 | 112.79 |
| 130 | W1559 | Thailand | 15 | 100 | 353 | W3006 | China | 22.38 | 112.69 |
| 131 | W1560 | Thailand | 14.03 | 100.37 | 354 | W3007 | China | 22.38 | 112.69 |
| 132 | W1619 | Thailand | 14.5 | 100.89 | 355 | W3008 | China | 22.53 | 113.04 |
| 133 | W1666 | India | 26.34 | 89.03 | 356 | W3009 | China | 22.89 | 112.85 |
| 134 | W1668 | India | 18.52 | 82.46 | 357 | W3010 | China | 23.17 | 112.89 |
| 135 | W1669 | India | 19.08 | 82.45 | 358 | W3011 | China | 23.17 | 112.89 |
| 136 | W1675 | India | 20.29 | 86.01 | 359 | W3012 | China | 23.12 | 113.26 |
| 137 | W1676 | India | 20.29 | 86.01 | 360 | W3013 | China | 23.12 | 113.26 |
| 138 | W1677 | India | 20.29 | 86.01 | 361 | W3014 | China | 23.29 | 113.83 |
| 139 | W1679 | India | 20.29 | 86.01 | 362 | W3015 | China | 23.29 | 113.83 |
| 140 | W1681 | India | 20.09 | 84.45 | 363 | W3016 | China | 23.05 | 113.75 |
| 141 | W1683 | India | 20.1 | 84.48 | 364 | W3017 | China | 23.05 | 113.75 |
| 142 | W1685 | India | 20.14 | 85.47 | 365 | W3018 | China | 22.8 | 114.46 |
| 143 | W1687 | India | 23.04 | 88.17 | 366 | W3019 | China | 22.8 | 114.46 |
| 144 | W1690 | Thailand | 19.41 | 99.34 | 367 | W3020 | China | 23.17 | 112.89 |
| 145 | W1695 | Thailand | 19.45 | 99.44 | 368 | W3021 | China | 23.87 | 113.53 |
| 146 | W1696 | Thailand | 19.45 | 99.44 | 369 | W3022 | China | 23.72 | 113.02 |
| 147 | W1698 | Thailand | 14.34 | 100.59 | 370 | W3023 | China | 23.72 | 113.02 |
| 148 | W1700 | Thailand | 14.3 | 100.55 | 371 | W3024 | China | 23.87 | 113.53 |
| 149 | W1715 | China | NA | NA | 372 | W3025 | China | 23.72 | 113.02 |
| 150 | W1716 | China | NA | NA | 373 | W3026 | China | 23.64 | 115.17 |
| 151 | W1718 | China | NA | NA | 374 | W3027 | China | 21.95 | 108.61 |
| 152 | W1719 | China | NA | NA | 375 | W3028 | China | 21.95 | 108.61 |
| 153 | W1721 | China | NA | NA | 376 | W3029 | China | 21.8 | 109.19 |
| 154 | W1723 | China | NA | NA | 377 | W3030 | China | 21.8 | 109.19 |
| 155 | W1724 | China | NA | NA | 378 | W3031 | China | 21.77 | 108.36 |
| 156 | W1725 | Thailand | 15 | 100 | 379 | W3032 | China | 21.77 | 108.36 |
| 157 | W1726 | Thailand | 15 | 100 | 380 | W3033 | China | 22.28 | 109.97 |
| 158 | W1727 | Thailand | 15 | 100 | 381 | W3034 | China | 22.28 | 109.97 |
| 159 | W1731 | India | 20.46 | 85.88 | 382 | W3035 | China | 22.63 | 110.14 |
| 160 | W1732 | India | 19.5 | 84.81 | 383 | W3036 | China | 22.63 | 110.14 |
| 161 | W1735 | India | 26.92 | 75.82 | 384 | W3037 | China | 23.07 | 109.36 |
| 162 | W1736 | India | 26.92 | 75.82 | 385 | W3038 | China | 23.07 | 109.36 |
| 163 | W1737 | India | 26.92 | 75.82 | 386 | W3039 | China | 23.39 | 110.07 |
| 164 | W1738 | India | 26.92 | 75.82 | 387 | W3040 | China | 23.39 | 110.07 |
| 165 | W1739 | India | 26.92 | 75.82 | 388 | W3041 | China | 22.75 | 108.49 |
| 166 | W1740 | India | 26.92 | 75.82 | 389 | W3042 | China | 22.75 | 108.49 |
| 167 | W1741 | India | 26.92 | 75.82 | 390 | W3043 | China | 23.17 | 108.28 |
| 168 | W1742 | India | 26.92 | 75.82 | 391 | W3044 | China | 23.17 | 108.28 |
| 169 | W1743 | India | 26.92 | 75.82 | 392 | W3045 | China | 22.69 | 109.27 |
| 170 | W1746 | India | 26.92 | 75.82 | 393 | W3046 | China | 22.69 | 109.27 |
| 171 | W1747 | India | 26.92 | 75.82 | 394 | W3047 | China | 23.73 | 106.91 |
| 172 | W1748 | India | 26.92 | 75.82 | 395 | W3048 | China | 23.9 | 106.61 |
| 173 | W1749 | India | 18.4 | 81.68 | 396 | W3049 | China | 23.9 | 106.61 |
| 174 | W1750 | India | 18.4 | 81.68 | 397 | W3050 | China | 23.9 | 106.61 |
| 175 | W1751 | India | 18.4 | 81.68 | 398 | W3051 | China | 23.9 | 106.61 |
| 176 | W1753 | India | 18.4 | 81.68 | 399 | W3052 | China | 23.73 | 106.91 |
| 177 | W1754 | India | 20.27 | 81.5 | 400 | W3053 | China | 19.1 | 110.35 |
| 178 | W1756 | India | 20.27 | 81.5 | 401 | W3054 | China | 19.1 | 110.35 |
| 179 | W1757 | India | 20.27 | 81.5 | 402 | W3055 | China | 19.1 | 110.35 |
| 180 | W1759 | India | 20.27 | 81.5 | 403 | W3056 | China | 19.5 | 109.5 |
| 181 | W1761 | India | 21.25 | 81.63 | 404 | W3057 | China | 19.5 | 109.5 |
| 182 | W1762 | India | 21.25 | 81.63 | 405 | W3058 | China | 19.1 | 109 |
| 183 | W1766 | India | 21.25 | 81.63 | 406 | W3059 | China | 19.1 | 109 |
| 184 | W1770 | India | 21.18 | 81.36 | 407 | W3060 | China | 18.75 | 109.17 |
| 185 | W1777 | India | 19.95 | 79.3 | 408 | W3061 | China | 18.75 | 109.17 |
| 186 | W1780 | India | 17 | 81.8 | 409 | W3062 | China | 19.1 | 109 |
| 187 | W1782 | India | 12.31 | 76.64 | 410 | W3063 | China | 19.1 | 109 |
| 188 | W1783 | India | 12.36 | 76.63 | 411 | W3064 | China | 19.25 | 110.46 |
| 189 | W1784 | India | 12.31 | 76.66 | 412 | W3065 | China | 19.25 | 110.46 |
| 190 | W1787 | Thailand | 15 | 100 | 413 | W3066 | China | 18.75 | 109.17 |
| 191 | W1788 | Thailand | 15 | 100 | 414 | W3067 | China | 18.65 | 109.8 |
| 192 | W1790 | Thailand | 15 | 100 | 415 | W3068 | China | 18.65 | 109.8 |
| 193 | W1792 | Thailand | 15 | 100 | 416 | W3069 | China | 18.65 | 109.8 |
| 194 | W1794 | Thailand | 15 | 100 | 417 | W3070 | China | 18.65 | 109.8 |
| 195 | W1795 | Thailand | 15 | 100 | 418 | W3071 | China | 19.62 | 110.7 |
| 196 | W1798 | Thailand | 15 | 100 | 419 | W3072 | China | 19.62 | 110.7 |
| 197 | W1802 | Bangladesh | 23.71 | 90.41 | 420 | W3073 | China | 19.62 | 110.7 |
| 198 | W1803 | Sri Lanka | 6.93 | 79.95 | 421 | W3074 | China | 19.62 | 110.7 |
| 199 | W1804 | Sri Lanka | 6.93 | 79.95 | 422 | W3075 | China | 28.23 | 116.61 |
| 200 | W1806 | Sri Lanka | 6.93 | 79.95 | 423 | W3076 | China | 28.23 | 116.61 |
| 201 | W1807 | Sri Lanka | 6.93 | 79.95 | 424 | W3077 | China | 28.23 | 116.61 |
| 202 | W1809 | Sri Lanka | 6.93 | 79.95 | 425 | W3078 | China | 28.23 | 116.61 |
| 203 | W1810 | Sri Lanka | 6.93 | 79.95 | 426 | W3079 | China | 28.23 | 116.61 |
| 204 | W1811 | Sri Lanka | 6.93 | 79.95 | 427 | W3080 | China | 28.23 | 116.61 |
| 205 | W1813 | Sri Lanka | 6.93 | 79.95 | 428 | W3081 | China | 28.23 | 116.61 |
| 206 | W1818 | Bangladesh | 23.71 | 90.41 | 429 | W3082 | China | 28.23 | 116.61 |
| 207 | W1819 | Bangladesh | 23.71 | 90.41 | 430 | W3083 | China | 22.1 | 100.79 |
| 208 | W1820 | Bangladesh | 23.71 | 90.41 | 431 | W3084 | China | 22.1 | 100.79 |
| 209 | W1821 | Bangladesh | 23.71 | 90.41 | 432 | W3085 | China | 23.6 | 102.01 |
| 210 | W1822 | Bangladesh | 23.71 | 90.41 | 433 | W3086 | China | 23.6 | 102.01 |
| 211 | W1823 | Bangladesh | 23.71 | 90.41 | 434 | W3087 | China | 23.6 | 102.01 |
| 212 | W1824 | Bangladesh | 23.71 | 90.41 | 435 | W3088 | China | 23.6 | 102.01 |
| 213 | W1825 | Bangladesh | 23.71 | 90.41 | 436 | W3089 | China | 22.1 | 100.79 |
| 214 | W1832 | Thailand | 13.92 | 100.59 | 437 | W3090 | China | 22.1 | 100.79 |
| 215 | W1839 | China | NA | NA | 438 | W3091 | China | 26.8 | 113.55 |
| 216 | W1844 | China | NA | NA | 439 | W3092 | China | 26.8 | 113.55 |
| 217 | W1849 | Thailand | 19.56 | 99.7 | 440 | W3093 | China | 25.28 | 111.34 |
| 218 | W1850 | Thailand | 19.56 | 99.7 | 441 | W3094 | China | 25.28 | 111.34 |
| 219 | W1852 | Thailand | 20.28 | 100.09 | 442 | W3095 | China | 25.28 | 111.34 |
| 220 | W1853 | Thailand | 19.75 | 99.73 | 443 | W3096 | China | 25.28 | 111.34 |
| 221 | W1854 | Thailand | 19.64 | 99.52 | 444 | W3097 | China | 25.28 | 111.34 |
| 222 | W1857 | Thailand | 18.35 | 99.53 | 445 | W3098 | China | 25.28 | 111.34 |
| 223 | W1858 | Thailand | 17.53 | 100.12 | 446 | W3105 | India | NA | NA |

Reference:

Huang, X.H., Kurata, N., Wei, X.H., Wang, Z.X., Wang, A.H., Zhao, Q., et al. (2012). A map of rice genome variation reveals the origin of cultivated rice. *Nature* 490, 497–501. doi:10.1038/nature11532
