# Supplementary material for: A new allele PEL9 GG identified by genome-wide association study increases panicle elongation length in rice (Oryza sativa L.)
Source: Front Plant Sci. 2023 Feb 16;14:1136549. doi: 10.3389/fpls.2023.1136549 (PMC9978329; doi:10.3389/fpls.2023.1136549)
Supplement: Supplementary file 10 [file Table_8.doc]

**Table S8.** SNP distribution of *PEL9* in 446 Oryza rufipogon reported by Huang et al. (2012).

| Code | ID name | SNP distribution | | | | | | Code | ID name | SNP distribution | | | | | |
| --- | --- | --- | --- | --- | --- | --- | --- | --- | --- | --- | --- | --- | --- | --- | --- |
|  |  | *PEL9*-LOC_Os09g18390 | | | | | |  |  | *PEL9*-LOC_Os09g18390 | | | | | |
|  |  | 11,289,927 | 11,290,285 | 11,290,484 | 11,290,715 | 11,290,777 | 11,291,449 |  |  | 11,289, 927 | 11,290,285 | 11,290,484 | 11,290,715 | 11,290,777 | 11,291,449 |
| 1 | W0101 | G | T | T | G | T | C | 224 | W1896 | G | T | T | G | T | T |
| 2 | W0102 | G | T | T | G | T | C | 225 | W1916 | G | T | T | G | T | C |
| 3 | W0103 | G | T | T | G | T | C | 226 | W1919 | G | T | T | G | T | C |
| 4 | W0106 | G | C | T | G | T | C | 227 | W1927 | G | T | A | A | T | T |
| 5 | W0107 | G | C | T | G | T | C | 228 | W1939 | G | T | T | G | T | T |
| 6 | W0121 | G | C | T | G | T | C | 229 | W1940 | G | T | T | G | T | C |
| 7 | W0123 | G | C | T | G | T | C | 230 | W1971 | G | C | T | G | T | T |
| 8 | W0124 | G | C | T | G | T | C | 231 | W1972 | G | T | A | A | G | T |
| 9 | W0128 | G | C | T | G | T | T | 232 | W1974 | G | C | T | G | T | T |
| 10 | W0130 | G | T | T | G | T | C | 233 | W1975 | G | C | T | G | T | C |
| 11 | W0144 | G | C | T | G | T | C | 234 | W1976 | G | C | T | G | T | C |
| 12 | W0147 | G | C | T | G | T | C | 235 | W1977 | G | C | A | A | G | C |
| 13 | W0148 | G | C | T | G | T | C | 236 | W1978 | G | C | T | G | T | T |
| 14 | W0151 | G | T | T | G | T | C | 237 | W1979 | G | C | T | G | T | T |
| 15 | W0152 | G | C | T | G | T | C | 238 | W1981 | G | T | T | G | T | C |
| 16 | W0168 | G | C | T | G | T | C | 239 | W2017 | G | C | T | G | T | T |
| 17 | W0170 | G | C | T | G | T | C | 240 | W2021 | G | T | A | A | G | T |
| 18 | W0173 | G | C | T | G | T | C | 241 | W2022 | G | C | T | G | T | C |
| 19 | W0176 | G | C | T | G | T | T | 242 | W2024 | G | T | A | A | T | T |
| 20 | W0178 | G | C | T | G | T | C | 243 | W2025 | G | C | T | G | T | C |
| 21 | W0179 | G | C | T | G | T | C | 244 | W2030 | G | T | A | A | T | T |
| 22 | W0574 | G | T | T | G | T | C | 245 | W2050 | G | C | T | G | T | C |
| 23 | W0589 | G | T | T | G | T | C | 246 | W2051 | G | C | T | G | T | T |
| 24 | W0590 | G | T | A | A | T | T | 247 | W2052 | G | C | T | G | T | T |
| 25 | W0605 | G | C | T | G | T | C | 248 | W2055 | G | C | T | G | T | T |
| 26 | W0610 | G | T | T | G | T | C | 249 | W2056 | G | C | T | G | T | C |
| 27 | W0626 | G | C | T | G | T | C | 250 | W2197 | G | C | T | G | T | T |
| 28 | W0627 | G | C | T | G | T | C | 251 | W2264 | G | C | T | G | T | C |
| 29 | W0630 | G | C | T | G | T | C | 252 | W2266 | G | C | T | G | T | T |
| 30 | W0631 | G | C | T | G | T | C | 253 | W2272 | G | C | T | G | T | C |
| 31 | W0632 | G | T | T | G | T | C | 254 | W2276 | G | C | T | G | T | C |
| 32 | W0633 | G | C | T | G | T | C | 255 | W2282 | G | T | T | G | T | C |
| 33 | W0638 | G | C | T | G | T | C | 256 | W2283 | G | T | A | G | T | C |
| 34 | W0639 | G | T | T | G | T | C | 257 | W2284 | G | C | T | G | T | C |
| 35 | W1080 | G | T | T | G | T | C | 258 | W2288 | G | T | A | G | T | T |
| 36 | W1082 | G | C | T | G | T | C | 259 | W2308 | G | C | T | G | T | T |
| 37 | W1083 | G | C | T | G | T | C | 260 | W2310 | G | C | T | G | T | C |
| 38 | W1084 | G | C | T | G | T | C | 261 | W2311 | G | T | T | G | T | C |
| 39 | W1086 | G | T | T | G | T | C | 262 | W2318 | G | C | T | G | T | T |
| 40 | W1090 | G | T | T | G | T | C | 263 | W2320 | G | C | T | G | T | C |
| 41 | W1092 | G | T | T | G | T | C | 264 | W2321 | G | C | T | G | T | C |
| 42 | W1105 | G | T | T | G | T | T | 265 | W2322 | G | C | T | G | T | C |
| 43 | W1107 | G | T | T | G | T | C | 266 | W2327 | G | C | T | G | T | T |
| 44 | W1111 | G | C | T | G | T | C | 267 | W3003 | G | C | T | G | T | C |
| 45 | W1112 | G | T | T | G | T | C | 268 | W3067 | G | C | T | G | T | C |
| 46 | W1117 | G | T | T | G | T | C | 269 | W3068 | G | C | T | G | T | T |
| 47 | W1121 | G | T | T | G | T | C | 270 | W3072 | G | T | T | G | T | T |
| 48 | W1142 | G | C | T | G | T | C | 271 | W3084 | G | T | T | G | G | C |
| 49 | W1143 | G | C | T | G | T | C | 272 | W3089 | G | T | A | G | T | C |
| 50 | W1230 | G | T | T | G | T | C | 273 | W3090 | G | C | T | G | T | C |
| 51 | W1295 | G | C | T | G | T | C | 274 | W3091 | G | C | T | G | T | C |
| 52 | W1532 | G | C | T | G | T | C | 275 | W3097 | G | T | T | G | G | T |
| 53 | W1533 | G | C | T | G | T | C | 276 | W3098 | G | T | A | G | G | T |
| 54 | W1546 | G | C | T | G | T | T | 277 | W0125 | G | T | T | G | T | C |
| 55 | W1547 | G | C | T | G | T | T | 278 | W0126 | G | T | T | G | T | C |
| 56 | W1551 | G | C | T | G | T | T | 279 | W0133 | G | T | A | G | T | C |
| 57 | W1559 | G | T | T | G | T | C | 280 | W0134 | G | T | T | G | T | C |
| 58 | W1619 | G | C | T | G | T | C | 281 | W0135 | G | T | T | G | T | C |
| 59 | W1666 | G | C | T | G | T | C | 282 | W0136 | G | T | T | G | T | C |
| 60 | W1675 | G | C | T | G | T | C | 283 | W0137 | G | T | T | G | T | C |
| 61 | W1676 | G | C | T | G | T | C | 284 | W0138 | G | T | T | G | T | C |
| 62 | W1677 | G | T | T | G | T | C | 285 | W0141 | G | T | T | G | T | C |
| 63 | W1679 | G | C | T | G | T | C | 286 | W0149 | G | T | A | A | T | C |
| 64 | W1681 | G | T | T | G | T | C | 287 | W0153 | G | T | T | G | T | C |
| 65 | W1685 | G | C | T | G | T | C | 288 | W0172 | G | T | T | G | T | C |
| 66 | W1690 | G | C | T | G | T | C | 289 | W0549 | G | T | T | G | T | C |
| 67 | W1695 | G | C | T | G | T | C | 290 | W0573 | G | T | T | A | T | C |
| 68 | W1696 | G | C | T | G | T | C | 291 | W0593 | G | T | T | G | T | C |
| 69 | W1698 | G | C | T | G | T | T | 292 | W0621 | G | T | T | G | T | C |
| 70 | W1700 | G | C | T | G | T | C | 293 | W0623 | G | C | T | G | T | C |
| 71 | W1719 | G | T | T | G | T | C | 294 | W0625 | G | T | T | G | T | C |
| 72 | W1721 | G | T | T | G | T | T | 295 | W0629 | G | T | T | G | T | C |
| 73 | W1723 | G | T | T | G | T | C | 296 | W0637 | G | T | T | G | T | C |
| 74 | W1726 | G | C | T | G | T | C | 297 | W1102 | G | T | T | G | T | C |
| 75 | W1727 | G | C | T | G | T | C | 298 | W1114 | G | T | T | G | T | C |
| 76 | W1731 | G | C | T | G | T | C | 299 | W1119 | G | C | T | G | T | C |
| 77 | W1735 | G | T | T | G | T | C | 300 | W1161 | G | T | T | A | T | C |
| 78 | W1737 | G | C | T | G | T | C | 301 | W1214 | G | C | T | G | T | T |
| 79 | W1738 | G | C | T | G | T | C | 302 | W1238 | G | C | T | G | T | C |
| 80 | W1740 | G | T | T | G | T | C | 303 | W1244 | G | C | T | G | T | C |
| 81 | W1741 | G | T | T | G | T | C | 304 | W1294 | G | T | T | G | T | C |
| 82 | W1743 | G | T | T | G | T | C | 305 | W1534 | G | C | T | G | T | C |
| 83 | W1747 | G | T | T | G | T | C | 306 | W1536 | G | T | T | G | T | C |
| 84 | W1749 | G | T | T | G | T | C | 307 | W1542 | G | C | T | G | T | C |
| 85 | W1750 | G | T | T | G | T | C | 308 | W1560 | G | C | T | G | T | C |
| 86 | W1751 | G | T | T | G | T | C | 309 | W1668 | G | C | T | G | T | T |
| 87 | W1753 | G | C | T | G | T | C | 310 | W1669 | G | T | T | G | T | C |
| 88 | W1754 | G | C | T | G | T | C | 311 | W1716 | G | C | A | A | G | C |
| 89 | W1756 | G | C | T | G | T | C | 312 | W1724 | G | T | T | G | T | C |
| 90 | W1757 | G | C | T | G | T | C | 313 | W1725 | G | T | T | G | T | C |
| 91 | W1761 | G | C | T | G | T | C | 314 | W1732 | G | T | T | G | T | C |
| 92 | W1762 | G | C | T | G | T | C | 315 | W1739 | G | T | T | G | T | C |
| 93 | W1770 | G | C | T | G | T | C | 316 | W1746 | G | C | T | G | T | C |
| 94 | W1787 | G | C | T | G | T | C | 317 | W1748 | G | T | T | G | T | C |
| 95 | W1788 | G | C | T | G | T | T | 318 | W1759 | G | T | T | G | T | C |
| 96 | W1792 | G | T | T | G | T | C | 319 | W1766 | G | T | T | G | T | C |
| 97 | W1794 | G | T | T | G | T | C | 320 | W1777 | G | C | T | G | T | C |
| 98 | W1795 | G | C | T | G | T | T | 321 | W1782 | G | T | A | A | T | T |
| 99 | W1802 | G | C | T | G | T | C | 322 | W1783 | G | C | T | G | T | C |
| 100 | W1806 | G | T | T | G | T | C | 323 | W1784 | G | C | T | G | T | C |
| 101 | W1818 | G | C | T | G | T | C | 324 | W1803 | G | T | T | G | T | C |
| 102 | W1819 | G | T | T | G | T | C | 325 | W1807 | G | T | A | A | T | T |
| 103 | W1820 | G | C | T | G | T | C | 326 | W1809 | G | T | T | G | T | C |
| 104 | W1821 | G | C | T | G | T | T | 327 | W1811 | G | T | T | A | T | C |
| 105 | W1822 | G | C | T | G | T | C | 328 | W1813 | G | T | T | A | T | C |
| 106 | W1823 | G | C | T | G | T | T | 329 | W1844 | G | T | T | A | T | T |
| 107 | W1824 | G | C | T | G | T | C | 330 | W1943 | G | T | A | A | G | T |
| 108 | W1825 | G | T | A | A | T | T | 331 | W1945 | G | T | A | G | T | T |
| 109 | W1832 | G | C | T | G | T | C | 332 | W1952 | G | T | A | G | T | T |
| 110 | W1839 | G | T | T | G | T | C | 333 | W1957 | G | C | T | G | T | C |
| 111 | W1852 | G | T | A | A | G | T | 334 | W1958 | G | C | T | G | T | C |
| 112 | W1853 | G | T | T | G | G | C | 335 | W1963 | G | C | T | G | T | C |
| 113 | W1865 | G | C | T | G | T | C | 336 | W1973 | G | T | A | A | T | T |
| 114 | W1866 | G | C | T | G | T | C | 337 | W1989 | G | T | T | G | T | C |
| 115 | W1879 | G | C | T | G | T | C | 338 | W1990 | G | T | T | G | T | C |
| 116 | W1881 | G | C | T | G | T | C | 339 | W1991 | G | T | T | G | T | C |
| 117 | W1893 | G | T | T | G | T | T | 340 | W1993 | G | T | T | G | T | C |
| 118 | W1912 | G | C | T | G | T | T | 341 | W1995 | G | T | T | G | T | C |
| 119 | W1914 | G | C | T | G | T | T | 342 | W1998 | G | C | T | G | T | C |
| 120 | W1921 | G | C | T | G | T | C | 343 | W2003 | G | C | T | G | T | C |
| 121 | W1925 | G | C | T | G | T | T | 344 | W2005 | G | C | T | G | T | C |
| 122 | W1928 | G | T | T | G | T | C | 345 | W2007 | G | C | T | G | T | C |
| 123 | W1935 | G | T | T | G | T | C | 346 | W2008 | G | C | T | G | T | C |
| 124 | W1959 | G | C | T | G | T | C | 347 | W2010 | G | C | T | G | T | C |
| 125 | W1970 | G | C | T | G | T | C | 348 | W2012 | G | C | T | G | T | C |
| 126 | W1983 | G | C | T | G | T | C | 349 | W2014 | G | C | T | G | T | C |
| 127 | W2053 | G | C | T | G | T | T | 350 | W2036 | G | C | T | G | T | T |
| 128 | W2060 | G | C | T | G | T | C | 351 | W2057 | G | T | T | G | T | C |
| 129 | W2061 | G | C | T | G | T | C | 352 | W2066 | G | C | T | G | T | C |
| 130 | W2063 | G | C | T | G | T | C | 353 | W2078 | G | T | T | G | T | C |
| 131 | W2064 | G | C | T | G | T | C | 354 | W2099 | G | T | A | G | T | C |
| 132 | W2193 | G | T | T | G | T | C | 355 | W2108 | G | T | T | G | T | C |
| 133 | W2263 | G | T | T | G | T | C | 356 | W2198 | G | T | T | G | T | C |
| 134 | W2265 | G | C | T | G | T | T | 357 | W2267 | G | T | A | A | G | T |
| 135 | W2268 | G | T | T | G | T | C | 358 | W3000 | G | T | T | G | T | T |
| 136 | W2269 | G | C | T | G | T | C | 359 | W3001 | G | T | A | A | G | T |
| 137 | W2271 | G | C | T | G | T | C | 360 | W3002 | G | C | T | G | T | C |
| 138 | W2275 | G | C | T | G | T | C | 361 | W3004 | G | C | T | G | G | C |
| 139 | W2277 | G | C | T | G | T | C | 362 | W3005 | G | T | A | A | G | T |
| 140 | W2278 | G | C | T | G | T | T | 363 | W3006 | G | C | T | G | T | T |
| 141 | W2296 | G | C | T | G | T | C | 364 | W3007 | G | T | T | G | T | C |
| 142 | W2298 | G | C | T | G | T | T | 365 | W3008 | G | T | T | G | T | C |
| 143 | W2299 | G | T | T | G | T | T | 366 | W3009 | G | T | T | G | T | C |
| 144 | W2301 | G | C | T | G | T | T | 367 | W3010 | G | C | T | G | T | C |
| 145 | W2302 | G | C | T | G | T | C | 368 | W3011 | G | C | T | A | G | T |
| 146 | W2303 | G | C | T | G | T | C | 369 | W3012 | G | C | T | A | T | T |
| 147 | W2304 | G | T | T | G | T | T | 370 | W3013 | G | T | A | A | G | T |
| 148 | W2305 | G | T | A | G | T | T | 371 | W3014 | G | C | T | G | T | C |
| 149 | W2306 | G | C | T | G | T | T | 372 | W3015 | G | T | A | G | T | C |
| 150 | W2307 | G | C | T | G | T | T | 373 | W3016 | G | T | A | A | T | T |
| 151 | W2316 | G | C | T | G | T | C | 374 | W3017 | G | T | A | A | G | C |
| 152 | W2319 | G | C | T | G | T | C | 375 | W3018 | G | C | T | G | T | C |
| 153 | W2331 | G | C | T | G | T | C | 376 | W3019 | G | T | T | G | T | T |
| 154 | W2332 | G | C | T | G | T | C | 377 | W3020 | G | T | T | G | T | T |
| 155 | W3105 | G | T | T | G | T | C | 378 | W3021 | G | T | T | G | T | C |
| 156 | W0108 | G | C | T | G | T | C | 379 | W3022 | G | T | T | G | T | C |
| 157 | W0120 | G | C | T | G | T | C | 380 | W3023 | G | C | T | G | T | T |
| 158 | W0132 | G | T | T | G | T | C | 381 | W3024 | G | C | T | G | T | T |
| 159 | W0143 | G | C | T | G | T | C | 382 | W3025 | G | C | T | G | T | C |
| 160 | W0145 | G | C | T | G | T | T | 383 | W3026 | G | C | T | G | T | T |
| 161 | W0157 | G | C | T | G | T | C | 384 | W3027 | G | T | A | A | G | T |
| 162 | W0163 | G | T | A | A | G | T | 385 | W3028 | G | T | T | G | T | C |
| 163 | W0164 | G | C | T | G | T | C | 386 | W3029 | G | T | T | G | T | C |
| 164 | W0165 | G | C | T | G | T | C | 387 | W3030 | G | C | T | A | G | T |
| 165 | W0166 | G | C | T | G | T | T | 388 | W3031 | G | T | T | G | T | C |
| 166 | W0169 | G | T | A | A | G | T | 389 | W3032 | G | T | T | G | T | T |
| 167 | W0171 | G | T | T | G | T | C | 390 | W3033 | G | C | T | G | T | C |
| 168 | W0174 | G | C | T | A | T | C | 391 | W3034 | G | T | A | A | G | T |
| 169 | W0175 | G | C | T | A | T | T | 392 | W3035 | G | T | T | G | T | C |
| 170 | W0180 | G | C | T | G | T | T | 393 | W3036 | G | C | T | G | T | C |
| 171 | W0234 | G | T | A | A | G | C | 394 | W3037 | G | C | T | G | T | C |
| 172 | W0576 | G | T | A | G | T | T | 395 | W3038 | G | C | T | G | T | C |
| 173 | W0587 | G | C | T | G | T | T | 396 | W3039 | G | T | A | A | T | T |
| 174 | W0594 | G | C | T | G | T | C | 397 | W3040 | G | T | A | A | G | T |
| 175 | W0596 | G | C | T | G | T | T | 398 | W3041 | G | T | A | A | G | C |
| 176 | W0600 | G | C | T | G | T | C | 399 | W3042 | G | T | A | G | T | T |
| 177 | W0606 | G | C | T | G | T | T | 400 | W3043 | G | T | T | G | T | T |
| 178 | W0624 | G | C | T | G | T | T | 401 | W3044 | G | T | T | G | T | C |
| 179 | W0628 | G | T | T | G | T | C | 402 | W3045 | G | T | T | G | T | T |
| 180 | W0634 | G | T | A | G | G | T | 403 | W3046 | G | T | A | G | T | T |
| 181 | W0635 | G | T | T | G | T | C | 404 | W3047 | G | T | A | A | T | T |
| 182 | W1087 | G | C | T | G | T | T | 405 | W3048 | G | C | T | G | T | C |
| 183 | W1093 | G | C | T | G | T | T | 406 | W3049 | G | T | A | A | T | T |
| 184 | W1096 | G | T | T | G | T | C | 407 | W3050 | G | T | A | A | G | T |
| 185 | W1122 | G | C | T | G | T | C | 408 | W3051 | G | T | A | A | T | T |
| 186 | W1124 | G | C | T | G | T | C | 409 | W3052 | G | T | A | A | G | T |
| 187 | W1126 | G | C | T | G | T | C | 410 | W3053 | G | T | A | A | T | T |
| 188 | W1236 | G | C | T | G | T | T | 411 | W3054 | G | T | A | A | G | T |
| 189 | W1292 | G | C | T | G | T | C | 412 | W3055 | G | T | A | A | T | T |
| 190 | W1550 | G | C | T | G | T | C | 413 | W3056 | G | T | T | G | T | C |
| 191 | W1552 | G | C | T | G | T | T | 414 | W3057 | G | T | T | G | T | T |
| 192 | W1553 | G | C | T | G | T | C | 415 | W3058 | G | C | T | G | T | C |
| 193 | W1554 | G | C | T | G | T | C | 416 | W3059 | G | C | T | G | T | T |
| 194 | W1555 | G | C | T | G | T | C | 417 | W3060 | G | T | T | A | T | T |
| 195 | W1556 | G | T | T | G | T | C | 418 | W3061 | G | C | T | G | T | T |
| 196 | W1557 | G | T | T | A | T | C | 419 | W3062 | G | C | T | A | T | C |
| 197 | W1558 | G | T | A | A | T | T | 420 | W3063 | G | T | T | A | T | C |
| 198 | W1683 | G | T | T | G | T | C | 421 | W3064 | G | C | T | G | T | C |
| 199 | W1687 | G | T | T | G | T | C | 422 | W3065 | G | C | T | G | T | C |
| 200 | W1715 | G | T | T | G | T | C | 423 | W3066 | G | C | T | G | T | T |
| 201 | W1718 | G | T | T | G | T | C | 424 | W3069 | G | C | T | G | T | C |
| 202 | W1736 | G | T | T | G | T | C | 425 | W3070 | G | C | T | G | T | C |
| 203 | W1742 | G | T | T | G | T | C | 426 | W3071 | G | C | T | G | T | C |
| 204 | W1780 | G | C | T | G | T | C | 427 | W3073 | G | T | T | G | T | C |
| 205 | W1790 | G | C | T | G | T | T | 428 | W3074 | G | T | A | A | G | T |
| 206 | W1798 | G | C | A | G | T | C | 429 | W3075 | G | T | T | G | T | C |
| 207 | W1804 | G | C | T | G | T | C | 430 | W3076 | G | T | A | A | T | T |
| 208 | W1810 | G | C | T | G | T | T | 431 | W3077 | G | T | A | A | G | T |
| 209 | W1849 | G | C | T | G | T | T | 432 | W3078 | G | T | A | G | T | T |
| 210 | W1850 | G | C | T | G | T | C | 433 | W3079 | G | T | A | A | T | C |
| 211 | W1854 | G | T | A | A | G | T | 434 | W3080 | G | T | A | A | G | T |
| 212 | W1857 | G | C | T | G | T | T | 435 | W3081 | G | T | A | A | G | T |
| 213 | W1858 | G | C | T | G | T | C | 436 | W3082 | G | T | T | G | T | C |
| 214 | W1859 | G | C | T | G | T | C | 437 | W3083 | G | T | A | A | T | C |
| 215 | W1862 | G | C | T | A | T | C | 438 | W3085 | G | T | A | A | T | C |
| 216 | W1870 | G | C | T | G | T | C | 439 | W3086 | G | T | A | A | G | C |
| 217 | W1873 | G | C | T | G | T | C | 440 | W3087 | G | T | A | A | T | C |
| 218 | W1880 | G | C | T | G | T | T | 441 | W3088 | G | . | T | G | T | C |
| 219 | W1882 | G | C | T | G | T | T | 442 | W3092 | G | T | T | G | T | C |
| 220 | W1884 | G | C | A | A | G | C | 443 | W3093 | G | T | A | A | G | T |
| 221 | W1890 | G | C | T | G | T | T | 444 | W3094 | G | C | T | G | T | T |
| 222 | W1891 | G | T | T | A | G | C | 445 | W3095 | G | T | T | G | T | T |
| 223 | W1895 | G | T | T | G | T | T | 446 | W3096 | G | C | T | A | T | C |
