# Supplementary material for: A new allele PEL9 GG identified by genome-wide association study increases panicle elongation length in rice (Oryza sativa L.)
Source: Front Plant Sci. 2023 Feb 16;14:1136549. doi: 10.3389/fpls.2023.1136549 (PMC9978329; doi:10.3389/fpls.2023.1136549)
Supplement: Supplementary file 11 [file Table_9.doc]

**Table S9.** Base information of 392 *Oryza sativa* download from RiceVarMap v2.0.

| Code | Accession name | ID name | Subgroup | Accession No. | Original | Code | Accession name | ID name | Subgroup | Accession No. | Original |
| --- | --- | --- | --- | --- | --- | --- | --- | --- | --- | --- | --- |
| 1 | Zacaodao_13 | B049 | AUS | ERS470264 | Nepal | 197 | Chao_peuak_deng | IRIS_313-10652 | INDIII | ERS469472 | Laos |
| 2 | N_22 | C013 | AUS | SRR1239613 | Philippines | 198 | BONGOLON | IRIS_313-10684 | INDIII | ERS469503 | Malaysia |
| 3 | SUGA_PANKHA | IRIS_313-10927 | Aus | ERS469726 | Nepal | 199 | BUJANG | IRIS_313-10699 | INDIII | ERS469514 | Malaysia |
| 4 | BAMLA_SUFFAID_320 | IRIS_313-11020 | Aus | ERS469817 | Pakistan | 200 | CHANTEK_145 | IRIS_313-10700 | INDIII | ERS469515 | Malaysia |
| 5 | BEGMI_135 | IRIS_313-11024 | Aus | ERS469821 | Pakistan | 201 | ANAK_CHINA | IRIS_313-10706 | INDIII | ERS469521 | Malaysia |
| 6 | MOTIA | IRIS_313-11028 | Aus | ERS469826 | Pakistan | 202 | ANAK_DIDEK | IRIS_313-10707 | INDIII | ERS469522 | Malaysia |
| 7 | 34 | IRIS_313-11036 | Aus | ERS469835 | Pakistan | 203 | ERNEST | IRIS_313-10725 | INDIII | ERS469531 | Senegal |
| 8 | 421 | IRIS_313-11037 | Aus | ERS469836 | Pakistan | 204 | ATJEH | IRIS_313-10760 | INDIII | ERS469570 | Indonesia |
| 9 | AUS_171 | IRIS_313-11048 | Aus | ERS469848 | Bangladesh | 205 | BANDI | IRIS_313-10762 | INDIII | ERS469572 | Indonesia |
| 10 | AUS_219 | IRIS_313-11049 | Aus | ERS469849 | Bangladesh | 206 | ARC_10159 | IRIS_313-10855 | INDIII | ERS469658 | India |
| 11 | AUS_233 | IRIS_313-11050 | Aus | ERS469850 | Bangladesh | 207 | ANG_KANG_K.P. | IRIS_313-10898 | INDIII | ERS469742 | Cambodia |
| 12 | AUS_242 | IRIS_313-11051 | Aus | ERS469851 | Bangladesh | 208 | DAMNOEUB_POR_HING | IRIS_313-10900 | INDIII | ERS469764 | Cambodia |
| 13 | AUS_278 | IRIS_313-11052 | Aus | ERS469852 | Bangladesh | 209 | BADAL | IRIS_313-10971 | INDIII | ERS469773 | Bangladesh |
| 14 | AUS_282 | IRIS_313-11053 | Aus | ERS469853 | Bangladesh | 210 | BOROJYOT | IRIS_313-10983 | INDIII | ERS469787 | Bangladesh |
| 15 | AUS_295 | IRIS_313-11054 | Aus | ERS469854 | Bangladesh | 211 | BALIBUD | IRIS_313-10990 | INDIII | ERS469823 | Philippines |
| 16 | AUS_299 | IRIS_313-11055 | Aus | ERS469855 | Bangladesh | 212 | ASE_BAKKA_LOMPO | IRIS_313-10995 | INDIII | ERS469878 | Indonesia |
| 17 | AUS_301 | IRIS_313-11056 | Aus | ERS469857 | Bangladesh | 213 | BENGKA_KERSEK | IRIS_313-11043 | INDIII | ERS469842 | Malaysia |
| 18 | AUS_308 | IRIS_313-11057 | Aus | ERS469858 | Bangladesh | 214 | Chao_hai | IRIS_313-11071 | INDIII | ERS469873 | Laos |
| 19 | AUS_329 | IRIS_313-11058 | Aus | ERS469859 | Bangladesh | 215 | Chao_lep_nok | IRIS_313-11072 | INDIII | ERS469874 | Laos |
| 20 | AUS_344 | IRIS_313-11059 | Aus | ERS469860 | Bangladesh | 216 | CADUNG_SO_BLOC_1 | IRIS_313-11096 | INDIII | ERS469891 | Vietnam |
| 21 | ARC_14756 | IRIS_313-11272 | Aus | ERS470126 | India | 217 | ANBAW | IRIS_313-11126 | INDIII | ERS469924 | Myanmar |
| 22 | ARC_14901 | IRIS_313-11274 | Aus | ERS470148 | India | 218 | BKN7130-1017-2 | IRIS_313-11192 | INDIII | ERS469982 | Thailand |
| 23 | ARC_15129 | IRIS_313-11277 | Aus | ERS470065 | India | 219 | DICULA | IRIS_313-11196 | INDIII | ERS469986 | Philippines |
| 24 | ARC_13544 | IRIS_313-11298 | Aus | ERS470088 | India | 220 | ADR52 | IRIS_313-11252 | INDIII | ERS470047 | India |
| 25 | AMAKOYALI | IRIS_313-11595 | Aus | ERS468782 | India | 221 | ARC_10120 | IRIS_313-11292 | INDIII | ERS470081 | India |
| 26 | LATIJHABAR | IRIS_313-11629 | Aus | ERS468808 | Nepal | 222 | ALAMINOS | IRIS_313-11330 | INDIII | ERS470123 | Philippines |
| 27 | PODI_HEENATI | IRIS_313-9636 | Aus | ERS467788 | Sri Lanka | 223 | AMPIPIT | IRIS_313-11331 | INDIII | ERS470124 | Philippines |
| 28 | KURKARUPPAN | IRIS_313-9861 | Aus | ERS467913 | Sri Lanka | 224 | DINGRAS | IRIS_313-11334 | INDIII | ERS470125 | Philippines |
| 29 | WIR1391 | IRIS_313-9963 | Aus | ERS468348 | Sri Lanka | 225 | AMON_4 | IRIS_313-11404 | INDIII | ERS470196 | Bangladesh |
| 30 | ARC_11751 | IRIS-313-10869 | AUS | ERS469672 | India | 226 | APYO-DAW-GYI | IRIS_313-11405 | INDIII | ERS470197 | Myanmar |
| 31 | ARC_11777 | IRIS-313-10871 | AUS | ERS469675 | India | 227 | A_28-6 | IRIS_313-11406 | INDIII | ERS470198 | Myanmar |
| 32 | ARC_11822 | IRIS-313-10873 | AUS | ERS469677 | India | 228 | BATCHA_BHOG(SCENTED) | IRIS_313-11407 | INDIII | ERS470199 | India |
| 33 | ARC_12021 | IRIS-313-10875 | AUS | ERS469679 | India | 229 | C_28-16 | IRIS_313-11410 | INDIII | ERS470202 | Myanmar |
| 34 | ARC_12067 | IRIS-313-10876 | AUS | ERS469680 | India | 230 | DONRADAO | IRIS_313-11411 | INDIII | ERS470203 | Brazil |
| 35 | ARC_12101 | IRIS-313-10878 | AUS | ERS469682 | India | 231 | BANGBANG | IRIS_313-11472 | INDIII | ERS468684 | Philippines |
| 36 | DHAN_263 | IRIS-313-11025 | AUS | ERS469822 | Pakistan | 232 | AR_133 | IRIS_313-11493 | INDIII | ERS468704 | India |
| 37 | JHONA_101 | IRIS-313-11027 | AUS | ERS469825 | Pakistan | 233 | ADIALLO | IRIS_313-11523 | INDIII | ERS468724 | Senegal |
| 38 | MUSHKAN_340_A | IRIS-313-11029 | AUS | ERS469827 | Pakistan | 234 | DIAMBARANG | IRIS_313-11525 | INDIII | ERS468726 | Guinea |
| 39 | ASWINA_330 | W330 | AUS | SRR1240133 | Bangladesh | 235 | BANGLA | IRIS_313-11561 | INDIII | ERS468756 | Nepal |
| 40 | Aijiaonante | B060 | INDI | ERS470274 | China | 236 | BIJULI_BATI | IRIS_313-11563 | INDIII | ERS468758 | Nepal |
| 41 | Dongtingwanxian | B083 | INDI | ERS470295 | China | 237 | Aerjituo | B017 | TEJ | ERS470235 | Bulgaria |
| 42 | Gongju_73 | B092 | INDI | ERS470304 | China | 238 | Albania_Rice | B034 | TEJ | ERS470251 | Albania |
| 43 | Chengduai_3_ | B114 | INDI | ERS470324 | China | 239 | Nabated_A_Smar | B038 | TEJ | ERS470255 | Egypt |
| 44 | Baikehanhe | B147 | INDI | ERS470357 | China | 240 | YR196 | B055 | TEJ | ERS470269 | Australia |
| 45 | 71011 | B181 | INDI | ERS470386 | Australia | 241 | 76--1 | B143 | TEJ | ERS470353 | China |
| 46 | Chaoyangyihao_B | B249 | INDI | ERS470447 | China | 242 | Baigedao | B162 | TEJ | ERS470372 | China |
| 47 | Chengduai3hao | C037 | INDI | SRR1239637 | Sichuan | 243 | Keluoduo_B | B179 | TEJ | ERS470384 | France |
| 48 | Gongju73 | C039 | INDI | SRR1239639 | Yunnan | 244 | Annongwangeng_B | B250 | TEJ | ERS470448 | China |
| 49 | Bawangbian1 | C059 | INDI | SRR1239659 | Hubei | 245 | AnnongwangengB-1 | C017 | TEJ | SRR1239617 | Anhui |
| 50 | Dongtingwanxian | C060 | INDI | SRR1239660 | Hunan | 246 | Funingzipi | C023 | TEJ | SRR1239623 | Hebei |
| 51 | Baikehualuo | C127 | INDI | SRR1239727 | Guangdong | 247 | Longhuamaohu | C032 | TEJ | SRR1239632 | Hebei |
| 52 | ChaoyangyihaoB | C156 | INDI | SRR1239756 | Hunan | 248 | Yelicanghua | C035 | TEJ | SRR1239635 | Hebei |
| 53 | Baikezaohe | C184 | INDI | SRR1239784 | Hunan | 249 | Gaoyangdiandao | C082 | TEJ | SRR1239682 | Hebei |
| 54 | Gang_46B | CX10 | INDI | ERS470464 | China | 250 | chikenuo | C083 | TEJ | SRR1239683 | Fujian |
| 55 | Baoxuan_21 | CX328 | INDI | ERS470619 | China | 251 | Xiangnuo-2 | C093 | TEJ | SRR1239693 | Guizhou |
| 56 | DoDo | GP35 | INDI | ERR036639 | Philippine | 252 | Babaili | C101 | TEJ | SRR1239701 | Yunnan |
| 57 | ARC_7286 | GP60 | INDI | ERR036664 | India | 253 | Yuyannuo-1 | C103 | TEJ | SRR1239703 | Yunnan |
| 58 | Aoyu-314 | GP676 | INDI | ERR036919 | Japan | 254 | Hongkezhenuo | C106 | TEJ | SRR1239706 | Guizhou |
| 59 | Banshanxian | HP184 | INDI | ERR009637 | China | 255 | Ximaxian | C111 | TEJ | SRR1239711 | Yunnan |
| 60 | Dayeqing | HP186 | INDI | ERR009827 | China | 256 | Xiangnuo-1 | C130 | TEJ | SRR1239730 | Guizhou |
| 61 | Anqinggu | HP219 | INDI | ERR009537 | China | 257 | Wuzidui | C134 | TEJ | SRR1239734 | Yunnan |
| 62 | Dayezao | HP236 | INDI | ERR009430 | China | 258 | Cungunuo | C137 | TEJ | SRR1239737 | Guizhou |
| 63 | Changainuo | HP250 | INDI | ERR009844 | China | 259 | Lengshuigu | C138 | TEJ | SRR1239738 | Yunnan |
| 64 | Gedadao | HP257 | INDI | ERR009723 | China | 260 | Huangpinuo | C144 | TEJ | SRR1239744 | Yunnan |
| 65 | Dayemaozhan | HP261 | INDI | ERR009857 | China | 261 | Zhonghua11 | C149 | TEJ | SRR1239749 | Beijing |
| 66 | Bairizao-4 | HP274 | INDI | ERR009567 | China | 262 | Zhonghua8hao | C172 | TEJ | SRR1239772 | Beijing |
| 67 | 503 | HP295 | INDI | ERR009780 | China | 263 | Haobayong1 | C179 | TEJ | SRR1239779 | Yunnan |
| 68 | Gouyingdu | HP299 | INDI | ERR009774 | China | 264 | Feidongtangdao | C181 | TEJ | SRR1239781 | Anhui |
| 69 | Dayezao | HP322 | INDI | ERR009429 | China | 265 | Niankenuo | C187 | TEJ | SRR1239787 | Guizhou |
| 70 | Baihonggu | HP359 | INDI | ERR009802 | China | 266 | Yangkenuo | C188 | TEJ | SRR1239788 | Guizhou |
| 71 | Bangbanggu | HP362 | INDI | ERR009520 | China | 267 | Yuyannuo-2 | C196 | TEJ | SRR1239796 | Yunnan |
| 72 | Bairizao | HP365 | INDI | ERR009461 | China | 268 | Nongken_58 | CX212 | TEJ | ERS470541 | Japan |
| 73 | Baijiugu | HP373 | INDI | ERR009826 | China | 269 | 02428-IL | CX277 | TEJ | ERS470588 | China |
| 74 | Baimaguzi | HP377 | INDI | ERR009454 | China | 270 | 053A-3 | CX351 | TEJ | ERS470636 | China |
| 75 | Gaicaohuang-2 | HP380 | INDI | ERR009590 | China | 271 | Giza159 | CX58 | TEJ | ERS470711 | Egypt |
| 76 | Dengbaoqi | HP394 | INDI | ERR009885 | China | 272 | M401 | CX78 | TEJ | ERS470731 | United States |
| 77 | Aigansazhima | HP407 | INDI | ERR009516 | China | 273 | Giza_172 | GP545 | TEJ | ERR036793 | Egypt |
| 78 | Gaicao | HP410 | INDI | ERR009750 | China | 274 | Nonglin-100 | GP548 | TEJ | ERR036796 | Japan |
| 79 | Dazhangu | HP413 | INDI | ERR009659 | China | 275 | Nonglin-106 | GP549 | TEJ | ERR036797 | Japan |
| 80 | Gaochahe | HP414 | INDI | ERR009907 | China | 276 | Delta | GP565 | TEJ | ERR036813 | France |
| 81 | Baipigu | HP415 | INDI | ERR009457 | China | 277 | Panuoxiameika | GP566 | TEJ | ERR036814 | Argentina |
| 82 | Baixianzi | HP432 | INDI | ERR009566 | China | 278 | Roma | GP568 | TEJ | ERR036816 | Italy |
| 83 | Baihuazhan | HP434 | INDI | ERR009700 | China | 279 | Mira | GP581 | TEJ | ERR036828 | Bulgaria |
| 84 | Gaicaohuang | HP437 | INDI | ERR009588 | China | 280 | Rosa_Marchetti | GP661 | TEJ | ERR036904 | Italy |
| 85 | Dongzhouliu | HP441 | INDI | ERR009822 | China | 281 | Giza_159 | GP88 | TEJ | ERR036692 | Egypt |
| 86 | Baimiyahzouzao | HP444 | INDI | ERR009529 | China | 282 | Baidadu | HP116 | TEJ | ERR037146 | China |
| 87 | Banbiannuo-2 | HP447 | INDI | ERR009784 | China | 283 | Baigen | HP152 | TEJ | ERR037180 | China |
| 88 | Chuanda-1 | HP452 | INDI | ERR009761 | China | 284 | Baiguonuo-1 | HP65 | TEJ | ERR009444 | China |
| 89 | Gaicaozhan | HP460 | INDI | ERR009940 | China | 285 | SANT_ANDREA | IRIS_313-10014 | TEJ | ERS468352 | Italy |
| 90 | Baiguomaweizhan | HP471 | INDI | ERR009796 | China | 286 | DACHEONGBYEO | IRIS_313-10059 | TEJ | ERS468355 | South Korea |
| 91 | Baizaogu | HP480 | INDI | ERR009943 | China | 287 | KALIN | IRIS_313-10089 | TEJ | ERS468358 | Bulgaria |
| 92 | Anhuihe | HP485 | INDI | ERR009577 | China | 288 | CHALBYEO | IRIS_313-10092 | TEJ | ERS468359 | South Korea |
| 93 | Chongyangnuo | HP498 | INDI | ERR009532 | China | 289 | CHEONJUDO | IRIS_313-10093 | TEJ | ERS468360 | South Korea |
| 94 | 77-2087 | HP504 | INDI | ERR009702 | China | 290 | GALSAEGSSAL_SHAREI | IRIS_313-10124 | TEJ | ERS468429 | South Korea |
| 95 | Babaili | HP509 | INDI | ERR009807 | China | 291 | 250_KUNGANI_1 | IRIS_313-10564 | TEJ | ERS469422 | Japan |
| 96 | Banjiuyan | HP513 | INDI | ERR009918 | China | 292 | SRAU_SLAP | IRIS_313-10916 | TEJ | ERS469714 | Cambodia |
| 97 | Dishuigu | HP517 | INDI | ERR009703 | China | 293 | IAS22-8_PALMAR | IRIS_313-10967 | TEJ | ERS469769 | Brazil |
| 98 | Baikezao | HP518 | INDI | ERR009431 | China | 294 | 4583 | IRIS_313-11202 | TEJ | ERS469993 | China |
| 99 | Changjingchi | HP524 | INDI | ERR009479 | China | 295 | BAI_MANG_AI_ZHONG | IRIS_313-11571 | TEJ | ERS468765 | China |
| 100 | Baishazhan | HP538 | INDI | ERR009509 | China | 296 | 36037-1 | IRIS_313-11586 | TEJ | ERS468778 | China |
| 101 | Baimiwujuzhan | HP542 | INDI | ERR009557 | China | 297 | 91-382 | IRIS_313-11661 | TEJ | ERS468838 | Bhutan |
| 102 | Changmaozhan | HP545 | INDI | ERR009922 | China | 298 | DECHANGBYEO | IRIS_313-11689 | TEJ | ERS468864 | South Korea |
| 103 | Chima | HP548 | INDI | ERR009576 | China | 299 | CN1067 | IRIS_313-11702 | TEJ | ERS468901 | United States |
| 104 | Chuangnuo-2 | HP549 | INDI | ERR009888 | China | 300 | GYEONGSAN_1 | IRIS_313-12003 | TEJ | ERS469141 | South Korea |
| 105 | Bayuehzhong | HP557 | INDI | ERR009572 | China | 301 | PIEMONTE | IRIS_313-8032 | TEJ | ERS468502 | Italy |
| 106 | Changsuihe | HP563 | INDI | ERR009510 | China | 302 | LOMELLINO | IRIS_313-8033 | TEJ | ERS468538 | Italy |
| 107 | Ganhangunuo | HP606 | INDI | ERR009840 | China | 303 | HARRA | IRIS_313-8075 | TEJ | ERS468523 | Australia |
| 108 | Chengduhonggu | HP75 | INDI | ERR009744 | China | 304 | CAPATAZ | IRIS_313-8084 | TEJ | ERS468519 | Spain |
| 109 | Dumai | B029 | INDII | ERS470246 | India | 305 | CLOT | IRIS_313-8085 | TEJ | ERS468489 | Spain |
| 110 | BW_293-2 | B033 | INDII | ERS470250 | Sri lanka | 306 | FRANCES | IRIS_313-8087 | TEJ | ERS468497 | Spain |
| 111 | Dangyu5hao | C069 | INDII | SRR1239669 | Anhui | 307 | CHIPKA | IRIS_313-8112 | TEJ | ERS468531 | Bulgaria |
| 112 | 9311 | C148 | INDII | SRR1239748 | Hunan | 308 | JUBILIENI | IRIS_313-8119 | TEJ | ERS468517 | Bulgaria |
| 113 | IR42 | CX161 | INDII | ERS470526 | Philippines | 309 | PLOVDIV_22 | IRIS_313-8125 | TEJ | ERS468539 | Bulgaria |
| 114 | IR2061-522-6-9 | CX206 | INDII | ERS470536 | Philippines | 310 | PLOVDIV_24 | IRIS_313-8126 | TEJ | ERS468480 | Bulgaria |
| 115 | Bg90-2 | CX21 | INDII | ERS470538 | Sri Lanka | 311 | POLIZESTI_28 | IRIS_313-8127 | TEJ | ERS468482 | Bulgaria |
| 116 | IR06G113 | CX226 | INDII | ERS470550 | Philippines | 312 | PRECOZ_2_F_A | IRIS_313-8128 | TEJ | ERS468665 | Argentina |
| 117 | IR64-IL | CX230 | INDII | ERS470554 | China | 313 | RODINA | IRIS_313-8129 | TEJ | ERS468506 | Bulgaria |
| 118 | IR62266-42-6-2 | CX234 | INDII | ERS470558 | Philippines | 314 | SAEDINENIE | IRIS_313-8135 | TEJ | ERS468513 | Bulgaria |
| 119 | BW311-9 | CX238 | INDII | ERS470562 | Philippines | 315 | SELN_244_A6-20 | IRIS_313-8141 | TEJ | ERS468481 | Australia |
| 120 | 2004 | CX249 | INDII | ERS470570 | China | 316 | YRM_6-2 | IRIS_313-8164 | TEJ | ERS468544 | Australia |
| 121 | 829 | CX250 | INDII | ERS470572 | China | 317 | SAL_BUI_BAO | CX106 | TRJ | ERS470470 | Vietnam |
| 122 | Duo_57 | CX290 | INDII | ERS470600 | China | 318 | Giza14 | CX111 | TRJ | ERS470476 | Egypt |
| 123 | D15 | CX291 | INDII | ERS470601 | China | 319 | MOLOK | CX129 | TRJ | ERS470494 | Indonesia |
| 124 | 117 | CX314 | INDII | ERS470612 | China | 320 | AP423 | CX132 | TRJ | ERS470498 | India |
| 125 | IR64a | CX403 | INDII | ERS470689 | Philippines | 321 | Manauthukha | CX32 | TRJ | ERS470618 | Myanmar |
| 126 | IR58025B | CX44 | INDII | ERS470693 | Philippines | 322 | BD007 | CX352 | TRJ | ERS470637 | China |
| 127 | Dhan4 | CX45 | INDII | ERS470694 | India | 323 | Yunlu_102 | CX355 | TRJ | ERS470640 | China |
| 128 | IR6 | CX73 | INDII | ERS470726 | Philippines | 324 | Yunlu_103 | CX359 | TRJ | ERS470644 | China |
| 129 | BG304 | CX76 | INDII | ERS470729 | Sri Lanka | 325 | Haogelao | CX367 | TRJ | ERS470652 | China |
| 130 | Cs94 | CX79 | INDII | ERS470732 | Vietnam | 326 | NERICA_1 | CX371 | TRJ | ERS470657 | Africa |
| 131 | X21 | CX82 | INDII | ERS470735 | Vietnam | 327 | NERICA_2 | CX372 | TRJ | ERS470658 | Africa |
| 132 | X22 | CX83 | INDII | ERS470736 | Vietnam | 328 | NERICA_8 | CX373 | TRJ | ERS470659 | Africa |
| 133 | X23 | CX84 | INDII | ERS470737 | Vietnam | 329 | NERICA_9 | CX374 | TRJ | ERS470660 | Africa |
| 134 | Budda | CX97 | INDII | ERS470750 | India | 330 | IAC_25 | GP39 | TRJ | ERR036643 | Brazil |
| 135 | IR29 | GP10 | INDII | ERR036614 | Philippine | 331 | Vary_Lava_10 | GP5 | TRJ | ERR036610 | Madagascar |
| 136 | IR_11297-139-2-2 | GP100 | INDII | ERR036704 | IRRI | 332 | Arborio | GP503 | TRJ | ERR036752 | Italy |
| 137 | IR_11297-158-1-1 | GP101 | INDII | ERR036705 | IRRI | 333 | Lunonglinnuo-1 | GP504 | TRJ | ERR036753 | Japan |
| 138 | IR_13240-10-1 | GP102 | INDII | ERR036706 | IRRI | 334 | Lunonglinnuo-12 | GP505 | TRJ | ERR036754 | Japan |
| 139 | IR_13543-66 | GP105 | INDII | ERR036709 | IRRI | 335 | Juannou | GP506 | TRJ | ERR036755 | Japan |
| 140 | IR30 | GP11 | INDII | ERR036615 | Philippine | 336 | Libu | GP514 | TRJ | ERR036762 | Philippine |
| 141 | IR_2006-P12-12-2-R | GP111 | INDII | ERR036715 | IRRI | 337 | Sipde-k | GP515 | TRJ | ERR036763 | Philippine |
| 142 | 77-061 | GP120 | INDII | ERR036722 | Philippine | 338 | Meranay | GP516 | TRJ | ERR036764 | Philippine |
| 143 | IR_11248-23-3-2 | GP129 | INDII | ERR036731 | IRRI | 339 | Yanayanan_(3) | GP517 | TRJ | ERR036765 | Philippine |
| 144 | IR661 | GP13 | INDII | ERR036617 | Philippine | 340 | Payakan | GP518 | TRJ | ERR036766 | Philippine |
| 145 | IR_17494-32-3-1-1-3 | GP130 | INDII | ERR036732 | IRRI | 341 | Manik | GP521 | TRJ | ERR036769 | Indonesia |
| 146 | Suweon_287 | GP135 | INDII | ERR036736 | North Korea | 342 | KETAN_DUNI | GP687 | TRJ | ERR036930 | Indonesia |
| 147 | IR_15529-256-1 | GP136 | INDII | ERR036737 | IRRI | 343 | GAJO | GP688 | TRJ | ERR036931 | Indonesia |
| 148 | Suweon_290 | GP137 | INDII | ERR036738 | North Korea | 344 | Bonjo | GP689 | TRJ | ERR036932 | Indonesia |
| 149 | IR_15685-2-2-2-3 | GP138 | INDII | ERR036739 | IRRI | 345 | R.S | GP690 | TRJ | ERR036933 | Indonesia |
| 150 | IR25588-7-3-1 | GP139 | INDII | ERR036740 | IRRI | 346 | Pate_Blanc_S3 | GP7 | TRJ | ERR036611 | Ivory Coast |
| 151 | IR_789-98-2-3-2-2 | GP14 | INDII | ERR036618 | IRRI | 347 | IFUGAO_RICE | IRIS_313-10577 | TRJ | ERS469435 | Philippines |
| 152 | IR25924-51-2-3 | GP140 | INDII | ERR036741 | IRRI | 348 | PARAY_QIKUG_KABAYUH | IRIS_313-10578 | TRJ | ERS469436 | Philippines |
| 153 | Suweon_294 | GP144 | INDII | ERR036745 | North Korea | 349 | PARAY_KINARABAW | IRIS_313-10580 | TRJ | ERS469438 | Philippines |
| 154 | IR_1561-228-3-3 | GP15 | INDII | ERR036619 | IRRI | 350 | P-PEY_SIQAT | IRIS_313-10581 | TRJ | ERS469439 | Philippines |
| 155 | IR_1702-74-3-2 | GP16 | INDII | ERR036620 | IRRI | 351 | QINOGNAS | IRIS_313-10582 | TRJ | ERS469440 | Philippines |
| 156 | IR_4422-164-3-6 | GP28 | INDII | ERR036632 | IRRI | 352 | KETAN_PAPAH_AREN | IRIS_313-10740 | TRJ | ERS469548 | Indonesia |
| 157 | IR_4427-253-5-1 | GP29 | INDII | ERR036633 | IRRI | 353 | KETAN_PELERN | IRIS_313-10741 | TRJ | ERS469549 | Indonesia |
| 158 | IR_4432-84-3-1 | GP30 | INDII | ERR036634 | IRRI | 354 | SERAJU | IRIS_313-10743 | TRJ | ERS469551 | Indonesia |
| 159 | IR_5311-46-3 | GP31 | INDII | ERR036635 | IRRI | 355 | SIPON | IRIS_313-10744 | TRJ | ERS469552 | Indonesia |
| 160 | C | GP32 | INDII | ERR036636 | Philippine | 356 | SLOBOK | IRIS_313-10745 | TRJ | ERS469553 | Indonesia |
| 161 | BG90-2 | GP40 | INDII | ERR036644 | Sri Lanka | 357 | TJEMPO_KREMBUNG | IRIS_313-10746 | TRJ | ERS469554 | Indonesia |
| 162 | Suweon_289 | GP637 | INDII | ERR036880 | North Korea | 358 | TJOKRON | IRIS_313-10747 | TRJ | ERS469556 | Indonesia |
| 163 | Suweon_320 | GP639 | INDII | ERR036882 | North Korea | 359 | BALIK_SEMAH | IRIS_313-10761 | TRJ | ERS469571 | Indonesia |
| 164 | Suweon_295 | GP652 | INDII | ERR036895 | North Korea | 360 | CERE_KAWAT | IRIS_313-10765 | TRJ | ERS469573 | Indonesia |
| 165 | IR_9095-258-2 | GP71 | INDII | ERR036675 | IRRI | 361 | DJALAWARA | IRIS_313-10766 | TRJ | ERS469574 | Indonesia |
| 166 | IR_4707-123-3 | GP73 | INDII | ERR036677 | IRRI | 362 | DJANGGOT | IRIS_313-10767 | TRJ | ERS469575 | Indonesia |
| 167 | IR_2003-P7-4-2 | GP79 | INDII | ERR036683 | IRRI | 363 | GANDAMANA | IRIS_313-10770 | TRJ | ERS469578 | Indonesia |
| 168 | IR_4422-98-3-6-1 | GP80 | INDII | ERR036684 | IRRI | 364 | GOGO_RAJAPAN | IRIS_313-10771 | TRJ | ERS469579 | Indonesia |
| 169 | IR_4567-69-1-3 | GP81 | INDII | ERR036685 | IRRI | 365 | HAWARA_DJEDAH | IRIS_313-10773 | TRJ | ERS469581 | Indonesia |
| 170 | IR_4563-52-1-3-6 | GP84 | INDII | ERR036688 | IRRI | 366 | KABADOKA | IRIS_313-10776 | TRJ | ERS469584 | Indonesia |
| 171 | IR_10181-58-3-1 | GP93 | INDII | ERR036697 | IRRI | 367 | KETAN_LALER | IRIS_313-10780 | TRJ | ERS469588 | Indonesia |
| 172 | IR_8608-189-2-2-1-3 | GP94 | INDII | ERR036698 | IRRI | 368 | KETAN_MLANTING | IRIS_313-10781 | TRJ | ERS469589 | Indonesia |
| 173 | IR_9115-40-1-3 | GP95 | INDII | ERR036699 | IRRI | 369 | KETAN_OSOG | IRIS_313-10783 | TRJ | ERS469591 | Indonesia |
| 174 | IR_8608-253-5-3-2 | GP97 | INDII | ERR036701 | IRRI | 370 | KETAN_SAPI | IRIS_313-10784 | TRJ | ERS469592 | Indonesia |
| 175 | IR_9093-195-1 | GP98 | INDII | ERR036702 | IRRI | 371 | KETAN_SLAWI | IRIS_313-10785 | TRJ | ERS469593 | Indonesia |
| 176 | BW295-5 | IRIS_313-10002 | INDII | ERS467881 | Sri Lanka | 372 | LEMUNGSIR | IRIS_313-10788 | TRJ | ERS469597 | Indonesia |
| 177 | BR-IRGA-409 | IRIS_313-10161 | INDII | ERS468163 | Brazil | 373 | PULO | IRIS_313-10805 | TRJ | ERS469652 | Indonesia |
| 178 | ICTA_CRISPO_38 | IRIS_313-10274 | INDII | ERS467883 | Guatemala | 374 | RANTE | IRIS_313-10808 | TRJ | ERS469684 | Indonesia |
| 179 | 3210 | IRIS_313-10298 | INDII | ERS468301 | Sri Lanka | 375 | SAMPANG_KUNING | IRIS_313-10809 | TRJ | ERS469695 | Indonesia |
| 180 | INIAP_10 | IRIS_313-10314 | INDII | ERS468194 | Ecuador | 376 | SIDJANGGUK | IRIS_313-10815 | TRJ | ERS469617 | Indonesia |
| 181 | IR_75870-5-8-5-B-1 | IRIS_313-10385 | INDII | ERS468201 | Philippines | 377 | SIDJERO_GUNDIL | IRIS_313-10816 | TRJ | ERS469618 | Indonesia |
| 182 | IR_77390-1-6-4-19-1-B | IRIS_313-10392 | INDII | ERS468305 | Philippines | 378 | SLAMET | IRIS_313-10817 | TRJ | ERS469619 | Indonesia |
| 183 | IR_1813-694-2 | IRIS_313-11122 | INDII | ERS469920 | Philippines | 379 | BINARITOS | IRIS_313-10827 | TRJ | ERS469629 | Philippines |
| 184 | IR_4535-PP_23-6-8-1 | IRIS_313-11249 | INDII | ERS470043 | Philippines | 380 | IKOGAN | IRIS_313-10828 | TRJ | ERS469630 | Philippines |
| 185 | C_894-21 | B027 | INDIII | ERS470244 | Philippines | 381 | INAMBOG | IRIS_313-10829 | TRJ | ERS469631 | Philippines |
| 186 | Abang_Busur | GP628 | INDIII | ERR036871 | Indonesia | 382 | INAPORAONAN | IRIS_313-10830 | TRJ | ERS469633 | Philippines |
| 187 | BANTA_TIMA | IRIS_313-10045 | INDIII | ERS468148 | Gambia | 383 | MALANDI_2 | IRIS_313-10831 | TRJ | ERS469634 | Philippines |
| 188 | EX_EBOKOZURU | IRIS_313-10109 | INDIII | ERS468229 | Nigeria | 384 | MALASAY | IRIS_313-10832 | TRJ | ERS469635 | Philippines |
| 189 | CASIBON | IRIS_313-10441 | INDIII | ERS469358 | Philippines | 385 | KETAN_NANGKA | IRIS_313-10841 | TRJ | ERS469645 | Indonesia |
| 190 | BINOLAYANGUN | IRIS_313-10448 | INDIII | ERS469361 | Philippines | 386 | SUNGKAI | IRIS_313-10960 | TRJ | ERS469761 | Indonesia |
| 191 | ADT22 | IRIS_313-10519 | INDIII | ERS469393 | India | 387 | GINATOS | IRIS_313-10991 | TRJ | ERS469834 | Philippines |
| 192 | BANDANG_PUTIH_680 | IRIS_313-10525 | INDIII | ERS469437 | Indonesia | 388 | PILIT_TAPUL | IRIS_313-10992 | TRJ | ERS469845 | Philippines |
| 193 | BELLO | IRIS_313-10542 | INDIII | ERS469402 | India | 389 | LANNAB | IRIS_313-10993 | TRJ | ERS469856 | Philippines |
| 194 | C_34-13 | IRIS_313-10547 | INDIII | ERS469406 | Myanmar | 390 | PANGETAN | IRIS_313-10994 | TRJ | ERS469867 | Philippines |
| 195 | BONG_SEN | IRIS_313-10554 | INDIII | ERS469411 | Vietnam | 391 | LANDEO | IRIS_313-10999 | TRJ | ERS469795 | Indonesia |
| 196 | BAANYALOJOPOIHUN | IRIS_313-10576 | INDIII | ERS469434 | Sierra Leone | 392 | PAEDAI_SOBUDI | IRIS_313-11001 | TRJ | ERS469797 | Indonesia |
