# Supplementary material for: A new allele PEL9 GG identified by genome-wide association study increases panicle elongation length in rice (Oryza sativa L.)
Source: Front Plant Sci. 2023 Feb 16;14:1136549. doi: 10.3389/fpls.2023.1136549 (PMC9978329; doi:10.3389/fpls.2023.1136549)
Supplement: Supplementary file 12 [file Table_10.doc]

**Table S10.** SNP distribution of *PEL9* in 392 Oryza sativa download from RiceVarMap v2.0.

| Code | ID name | SNP distribution | | | | | | Code | ID name | SNP distribution | | | | | |
| --- | --- | --- | --- | --- | --- | --- | --- | --- | --- | --- | --- | --- | --- | --- | --- |
|  |  | *PEL9*-*LOC_Os09g18390* | | | | | |  |  | *PEL9*-*LOC_Os09g18390* | | | | | |
|  |  | 11,289,927 | 11,290,285 | 11,290,484 | 11,290,715 | 11,290,777 | 11,291,449 |  |  | 11,289,927 | 11,290,285 | 11,290,484 | 11,290,715 | 11,290,777 | 11,291,449 |
| 1 | B049 | G | T | T | G | T | C | 197 | IRIS_313-10652 | G | T | T | G | T | C |
| 2 | C013 | G | T | T | G | T | C | 198 | IRIS_313-10684 | G | C | T | A | G | T |
| 3 | IRIS_313-10927 | A | C | A | A | G | C | 199 | IRIS_313-10699 | A | C | A | A | G | C |
| 4 | IRIS_313-11020 | G | T | T | G | T | C | 200 | IRIS_313-10700 | G | C | T | A | G | T |
| 5 | IRIS_313-11024 | G | T | T | G | T | C | 201 | IRIS_313-10706 | A | C | A | A | G | C |
| 6 | IRIS_313-11028 | G | T | T | G | T | C | 202 | IRIS_313-10707 | G | C | T | A | G | C |
| 7 | IRIS_313-11036 | G | T | T | G | T | C | 203 | IRIS_313-10725 | G | T | T | G | T | C |
| 8 | IRIS_313-11037 | A | C | A | A | G | C | 204 | IRIS_313-10760 | A | C | A | A | G | C |
| 9 | IRIS_313-11048 | G | T | T | G | T | C | 205 | IRIS_313-10762 | G | T | T | G | T | C |
| 10 | IRIS_313-11049 | G | T | T | G | T | C | 206 | IRIS_313-10855 | G | T | T | G | T | C |
| 11 | IRIS_313-11050 | G | T | T | G | T | C | 207 | IRIS_313-10898 | G | C | A | A | G | C |
| 12 | IRIS_313-11051 | A | C | A | A | G | C | 208 | IRIS_313-10900 | G | T | T | G | T | C |
| 13 | IRIS_313-11052 | G | T | T | G | T | C | 209 | IRIS_313-10971 | G | T | T | G | T | C |
| 14 | IRIS_313-11053 | G | T | T | G | T | C | 210 | IRIS_313-10983 | G | T | T | G | T | C |
| 15 | IRIS_313-11054 | G | T | T | G | T | C | 211 | IRIS_313-10990 | A | C | A | A | G | C |
| 16 | IRIS_313-11055 | G | T | T | G | T | C | 212 | IRIS_313-10995 | G | T | T | G | T | C |
| 17 | IRIS_313-11056 | G | T | T | G | T | C | 213 | IRIS_313-11043 | G | C | T | A | G | T |
| 18 | IRIS_313-11057 | G | T | T | G | T | C | 214 | IRIS_313-11071 | A | C | A | A | G | C |
| 19 | IRIS_313-11058 | G | T | T | G | T | C | 215 | IRIS_313-11072 | A | C | A | A | G | C |
| 20 | IRIS_313-11059 | G | T | T | G | T | C | 216 | IRIS_313-11096 | G | T | T | G | T | C |
| 21 | IRIS_313-11272 | G | T | T | G | T | C | 217 | IRIS_313-11126 | G | T | T | G | T | C |
| 22 | IRIS_313-11274 | G | T | T | G | T | C | 218 | IRIS_313-11192 | G | T | T | G | T | C |
| 23 | IRIS_313-11277 | G | T | T | G | T | C | 219 | IRIS_313-11196 | A | C | A | A | G | C |
| 24 | IRIS_313-11298 | G | T | T | G | T | C | 220 | IRIS_313-11252 | G | T | T | G | T | C |
| 25 | IRIS_313-11595 | G | T | T | G | T | C | 221 | IRIS_313-11292 | G | T | T | G | T | C |
| 26 | IRIS_313-11629 | G | T | T | G | T | C | 222 | IRIS_313-11330 | A | C | A | A | G | C |
| 27 | IRIS_313-9636 | G | C | T | A | G | C | 223 | IRIS_313-11331 | G | T | T | G | T | C |
| 28 | IRIS_313-9861 | A | C | A | A | G | C | 224 | IRIS_313-11334 | G | T | T | G | T | C |
| 29 | IRIS_313-9963 | G | T | T | G | T | C | 225 | IRIS_313-11404 | G | T | T | G | T | C |
| 30 | IRIS_313-10869 | A | C | A | A | G | C | 226 | IRIS_313-11405 | G | T | T | G | T | C |
| 31 | IRIS_313-10871 | G | T | T | G | T | C | 227 | IRIS_313-11406 | G | T | T | G | T | C |
| 32 | IRIS_313-10873 | G | T | T | G | T | C | 228 | IRIS_313-11407 | A | C | A | A | G | C |
| 33 | IRIS_313-10875 | G | T | T | G | T | C | 229 | IRIS_313-11410 | G | T | T | G | T | C |
| 34 | IRIS_313-10876 | G | T | T | G | T | C | 230 | IRIS_313-11411 | G | T | T | G | T | C |
| 35 | IRIS_313-10878 | A | C | A | A | G | C | 231 | IRIS_313-11472 | A | C | A | A | G | C |
| 36 | IRIS_313-11025 | A | C | A | A | G | C | 232 | IRIS_313-11493 | G | C | T | A | G | T |
| 37 | IRIS_313-11027 | G | T | T | G | T | C | 233 | IRIS_313-11523 | G | T | T | G | T | C |
| 38 | IRIS_313-11029 | G | T | T | G | T | C | 234 | IRIS_313-11525 | G | T | T | G | T | C |
| 39 | W330 | G | T | T | G | T | C | 235 | IRIS_313-11561 | G | T | T | G | T | C |
| 40 | B060 | G | T | T | G | T | C | 236 | IRIS_313-11563 | G | T | T | G | T | C |
| 41 | B083 | G | T | T | G | T | C | 237 | B017 | G | T | A | A | G | T |
| 42 | B092 | G | T | T | G | T | C | 238 | B034 | G | T | A | A | G | T |
| 43 | B114 | G | T | T | G | T | C | 239 | B038 | G | T | A | A | G | T |
| 44 | B147 | G | T | T | G | T | C | 240 | B055 | G | T | A | A | G | T |
| 45 | B181 | G | T | T | G | T | C | 241 | B143 | G | T | A | A | G | T |
| 46 | B249 | G | T | T | G | T | C | 242 | B162 | A | C | A | A | G | C |
| 47 | C037 | G | T | T | G | T | C | 243 | B179 | G | T | A | A | G | T |
| 48 | C039 | G | T | T | G | T | C | 244 | B250 | A | C | A | A | G | C |
| 49 | C059 | A | C | A | A | G | C | 245 | C017 | G | T | A | A | G | T |
| 50 | C060 | G | T | T | G | T | C | 246 | C023 | G | T | A | A | G | T |
| 51 | C127 | G | T | T | G | T | C | 247 | C032 | G | T | A | A | G | T |
| 52 | C156 | G | T | T | G | T | C | 248 | C035 | G | T | A | A | G | T |
| 53 | C184 | A | C | A | A | G | C | 249 | C082 | G | T | A | A | G | T |
| 54 | CX10 | G | T | T | G | T | C | 250 | C083 | A | C | A | A | G | C |
| 55 | CX328 | G | T | T | G | T | C | 251 | C093 | G | T | A | A | G | T |
| 56 | GP35 | G | T | T | G | T | C | 252 | C101 | G | T | A | A | G | T |
| 57 | GP60 | G | T | T | G | T | C | 253 | C103 | A | C | A | A | G | C |
| 58 | GP676 | G | T | T | G | T | C | 254 | C106 | G | T | T | G | T | C |
| 59 | HP184 | A | C | A | A | G | C | 255 | C111 | G | T | A | A | G | T |
| 60 | HP186 | G | T | T | G | T | C | 256 | C130 | G | T | A | A | G | T |
| 61 | HP219 | G | T | T | G | T | C | 257 | C134 | G | T | A | A | G | T |
| 62 | HP236 | G | T | T | G | T | C | 258 | C137 | G | T | T | G | T | C |
| 63 | HP250 | G | T | T | G | T | C | 259 | C138 | G | T | A | A | G | T |
| 64 | HP257 | G | T | T | G | T | C | 260 | C144 | A | C | A | A | G | C |
| 65 | HP261 | G | T | T | G | T | C | 261 | C149 | A | C | A | A | G | C |
| 66 | HP274 | G | T | T | G | T | C | 262 | C172 | A | C | A | A | G | C |
| 67 | HP295 | G | T | T | G | T | C | 263 | C179 | G | T | A | A | G | T |
| 68 | HP299 | A | C | A | A | G | C | 264 | C181 | G | T | A | A | G | T |
| 69 | HP322 | A | C | A | A | G | C | 265 | C187 | G | T | T | G | T | C |
| 70 | HP359 | G | T | T | G | T | C | 266 | C188 | G | T | A | A | G | T |
| 71 | HP362 | A | C | A | A | G | C | 267 | C196 | A | C | A | A | G | C |
| 72 | HP365 | A | C | A | A | G | C | 268 | CX212 | A | C | A | A | G | C |
| 73 | HP373 | A | C | A | A | G | C | 269 | CX277 | G | T | A | A | G | T |
| 74 | HP377 | G | T | T | G | T | C | 270 | CX351 | G | T | A | A | G | T |
| 75 | HP380 | G | T | T | G | T | C | 271 | CX58 | G | T | A | A | G | T |
| 76 | HP394 | G | T | T | G | T | C | 272 | CX78 | G | T | A | A | G | T |
| 77 | HP407 | G | T | T | G | T | C | 273 | GP545 | G | T | A | A | G | T |
| 78 | HP410 | G | T | T | G | T | C | 274 | GP548 | G | T | A | A | G | T |
| 79 | HP413 | G | T | T | G | T | C | 275 | GP549 | G | T | A | A | G | T |
| 80 | HP414 | G | T | T | G | T | C | 276 | GP565 | G | T | A | A | G | T |
| 81 | HP415 | G | T | T | G | T | C | 277 | GP566 | G | T | A | A | G | T |
| 82 | HP432 | G | T | T | G | T | C | 278 | GP568 | G | T | A | A | G | T |
| 83 | HP434 | G | T | T | G | T | C | 279 | GP581 | G | T | A | A | G | T |
| 84 | HP437 | G | T | T | G | T | C | 280 | GP661 | G | T | A | A | G | T |
| 85 | HP441 | G | T | T | G | T | C | 281 | GP88 | A | C | A | A | G | C |
| 86 | HP444 | G | T | T | G | T | C | 282 | HP116 | G | T | A | A | G | T |
| 87 | HP447 | G | T | T | G | T | C | 283 | HP152 | G | T | T | G | T | C |
| 88 | HP452 | G | T | T | G | T | C | 284 | HP65 | A | C | A | A | G | C |
| 89 | HP460 | G | T | T | G | T | C | 285 | IRIS_313-10014 | G | T | A | A | G | T |
| 90 | HP471 | A | C | A | A | G | C | 286 | IRIS_313-10059 | G | T | A | A | G | T |
| 91 | HP480 | G | T | T | G | T | C | 287 | IRIS_313-10089 | G | T | A | A | G | T |
| 92 | HP485 | G | T | T | G | T | C | 288 | IRIS_313-10092 | G | T | A | A | G | T |
| 93 | HP498 | A | C | A | A | G | C | 289 | IRIS_313-10093 | A | C | A | A | G | C |
| 94 | HP504 | G | T | T | G | T | C | 290 | IRIS_313-10124 | G | T | A | A | G | T |
| 95 | HP509 | G | T | T | G | T | C | 291 | IRIS_313-10564 | G | T | A | A | G | T |
| 96 | HP513 | A | C | A | A | G | C | 292 | IRIS_313-10916 | G | T | A | A | G | T |
| 97 | HP517 | G | T | T | G | T | C | 293 | IRIS_313-10967 | G | T | A | A | G | T |
| 98 | HP518 | A | C | A | A | G | C | 294 | IRIS_313-11202 | A | C | A | A | G | C |
| 99 | HP524 | G | T | T | G | T | C | 295 | IRIS_313-11571 | G | T | A | A | G | T |
| 100 | HP538 | A | C | A | A | G | C | 296 | IRIS_313-11586 | A | C | A | A | G | C |
| 101 | HP542 | G | T | T | G | T | C | 297 | IRIS_313-11661 | G | T | A | A | G | T |
| 102 | HP545 | G | T | T | G | T | C | 298 | IRIS_313-11689 | G | T | A | A | G | T |
| 103 | HP548 | G | T | T | G | T | C | 299 | IRIS_313-11702 | G | T | A | A | G | T |
| 104 | HP549 | G | T | T | G | T | C | 300 | IRIS_313-12003 | G | T | A | A | G | T |
| 105 | HP557 | G | T | T | G | T | C | 301 | IRIS_313-8032 | G | T | A | A | G | T |
| 106 | HP563 | G | T | T | G | T | C | 302 | IRIS_313-8033 | G | T | A | A | G | T |
| 107 | HP606 | G | T | T | G | T | C | 303 | IRIS_313-8075 | G | T | A | A | G | T |
| 108 | HP75 | G | T | T | G | T | C | 304 | IRIS_313-8084 | G | T | A | A | G | T |
| 109 | B029 | G | C | T | A | G | T | 305 | IRIS_313-8085 | G | T | A | A | G | T |
| 110 | B033 | G | T | T | G | T | C | 306 | IRIS_313-8087 | G | T | A | A | G | T |
| 111 | C069 | G | C | T | A | G | C | 307 | IRIS_313-8112 | G | T | A | A | G | T |
| 112 | C148 | G | T | T | G | T | C | 308 | IRIS_313-8119 | G | T | A | A | G | T |
| 113 | CX161 | G | T | T | G | T | C | 309 | IRIS_313-8125 | G | T | A | A | G | T |
| 114 | CX206 | G | T | T | A | G | T | 310 | IRIS_313-8126 | G | T | A | A | G | T |
| 115 | CX21 | G | C | T | A | G | C | 311 | IRIS_313-8127 | G | T | A | A | G | T |
| 116 | CX226 | G | T | T | G | T | C | 312 | IRIS_313-8128 | G | T | A | A | G | T |
| 117 | CX230 | G | T | T | G | T | C | 313 | IRIS_313-8129 | G | T | A | A | G | T |
| 118 | CX234 | G | T | T | G | T | C | 314 | IRIS_313-8135 | A | C | A | A | G | C |
| 119 | CX238 | G | T | T | G | T | C | 315 | IRIS_313-8141 | G | T | A | A | G | T |
| 120 | CX249 | A | C | A | A | G | C | 316 | IRIS_313-8164 | G | T | A | A | G | T |
| 121 | CX250 | A | C | A | A | G | C | 317 | CX106 | G | T | A | A | G | T |
| 122 | CX290 | A | C | A | A | G | C | 318 | CX111 | G | T | A | A | G | T |
| 123 | CX291 | A | C | A | A | G | C | 319 | CX129 | G | T | A | A | G | C |
| 124 | CX314 | G | T | A | A | G | T | 320 | CX132 | G | T | A | A | G | T |
| 125 | CX403 | G | T | T | G | T | C | 321 | CX32 | G | T | A | A | G | T |
| 126 | CX44 | G | T | T | G | T | C | 322 | CX352 | G | T | A | A | G | T |
| 127 | CX45 | G | C | T | A | G | C | 323 | CX355 | G | T | A | A | G | T |
| 128 | CX73 | G | T | T | G | T | C | 324 | CX359 | G | T | A | A | G | T |
| 129 | CX76 | G | T | T | G | T | C | 325 | CX367 | G | T | A | A | G | T |
| 130 | CX79 | G | T | T | G | T | C | 326 | CX371 | G | T | A | A | G | T |
| 131 | CX82 | G | T | T | G | T | C | 327 | CX372 | G | T | A | A | G | T |
| 132 | CX83 | G | C | T | A | G | C | 328 | CX373 | G | T | A | A | G | T |
| 133 | CX84 | G | T | T | G | T | C | 329 | CX374 | G | T | A | A | G | T |
| 134 | CX97 | G | T | T | G | T | C | 330 | GP39 | G | T | A | A | G | T |
| 135 | GP10 | G | C | T | A | G | T | 331 | GP5 | G | T | A | A | G | T |
| 136 | GP100 | G | C | T | A | G | C | 332 | GP503 | G | T | A | A | G | T |
| 137 | GP101 | G | T | T | G | T | C | 333 | GP504 | A | C | T | G | T | C |
| 138 | GP102 | G | T | T | G | T | C | 334 | GP505 | A | C | T | G | T | C |
| 139 | GP105 | G | T | T | G | T | C | 335 | GP506 | G | T | A | A | G | T |
| 140 | GP11 | G | T | T | G | T | C | 336 | GP514 | G | T | A | A | G | T |
| 141 | GP111 | G | T | T | G | T | C | 337 | GP515 | G | T | A | A | G | T |
| 142 | GP120 | G | T | T | G | T | C | 338 | GP516 | G | T | A | A | G | T |
| 143 | GP129 | G | T | T | G | T | C | 339 | GP517 | G | T | A | A | G | T |
| 144 | GP13 | G | T | T | G | T | C | 340 | GP518 | G | T | A | A | G | T |
| 145 | GP130 | G | T | T | G | T | C | 341 | GP521 | A | C | T | G | T | C |
| 146 | GP135 | G | C | T | A | G | C | 342 | GP687 | G | T | A | G | T | C |
| 147 | GP136 | G | T | T | G | T | C | 343 | GP688 | G | T | A | A | G | T |
| 148 | GP137 | G | C | T | A | G | T | 344 | GP689 | G | T | A | A | G | T |
| 149 | GP138 | G | T | T | G | T | C | 345 | GP690 | A | C | T | G | T | C |
| 150 | GP139 | G | T | T | G | T | C | 346 | GP7 | G | T | A | A | G | T |
| 151 | GP14 | G | T | T | G | T | C | 347 | IRIS_313-10577 | G | T | A | A | G | T |
| 152 | GP140 | G | T | T | G | T | C | 348 | IRIS_313-10578 | G | T | A | A | G | T |
| 153 | GP144 | G | T | T | G | T | C | 349 | IRIS_313-10580 | G | T | A | A | G | T |
| 154 | GP15 | G | T | T | G | T | C | 350 | IRIS_313-10581 | G | T | A | A | G | T |
| 155 | GP16 | G | T | T | G | T | C | 351 | IRIS_313-10582 | G | T | A | A | G | T |
| 156 | GP28 | G | T | T | G | T | C | 352 | IRIS_313-10740 | G | T | A | G | T | C |
| 157 | GP29 | G | T | T | G | T | C | 353 | IRIS_313-10741 | G | T | A | G | T | C |
| 158 | GP30 | G | T | T | G | T | C | 354 | IRIS_313-10743 | G | T | A | A | G | T |
| 159 | GP31 | G | T | T | G | T | C | 355 | IRIS_313-10744 | G | T | A | A | G | T |
| 160 | GP32 | G | T | T | G | T | C | 356 | IRIS_313-10745 | G | T | A | G | T | C |
| 161 | GP40 | G | C | T | A | G | C | 357 | IRIS_313-10746 | G | T | A | G | T | C |
| 162 | GP637 | G | T | T | G | T | C | 358 | IRIS_313-10747 | G | T | A | A | G | T |
| 163 | GP639 | G | T | T | G | T | C | 359 | IRIS_313-10761 | G | T | A | A | G | T |
| 164 | GP652 | G | T | T | G | T | C | 360 | IRIS_313-10765 | G | T | A | G | T | C |
| 165 | GP71 | G | T | T | G | T | C | 361 | IRIS_313-10766 | G | T | A | A | G | T |
| 166 | GP73 | G | C | T | A | G | T | 362 | IRIS_313-10767 | A | C | T | G | T | C |
| 167 | GP79 | G | T | T | G | T | C | 363 | IRIS_313-10770 | G | T | A | A | G | T |
| 168 | GP80 | G | C | T | A | G | T | 364 | IRIS_313-10771 | G | T | A | A | G | T |
| 169 | GP81 | G | T | T | G | T | C | 365 | IRIS_313-10773 | G | T | A | G | T | C |
| 170 | GP84 | G | T | T | G | T | C | 366 | IRIS_313-10776 | G | T | A | A | G | T |
| 171 | GP93 | G | T | T | G | T | C | 367 | IRIS_313-10780 | A | C | T | G | T | C |
| 172 | GP94 | G | T | T | G | T | C | 368 | IRIS_313-10781 | G | T | A | A | G | T |
| 173 | GP95 | G | T | T | G | T | C | 369 | IRIS_313-10783 | G | T | A | G | T | C |
| 174 | GP97 | G | T | T | G | T | C | 370 | IRIS_313-10784 | G | T | A | A | G | T |
| 175 | GP98 | G | T | T | G | T | C | 371 | IRIS_313-10785 | G | T | A | G | T | C |
| 176 | IRIS_313-10002 | G | T | T | G | T | C | 372 | IRIS_313-10788 | G | T | A | G | T | C |
| 177 | IRIS_313-10161 | G | T | T | G | T | C | 373 | IRIS_313-10805 | G | T | A | A | G | T |
| 178 | IRIS_313-10274 | G | C | T | A | G | C | 374 | IRIS_313-10808 | A | C | T | G | T | C |
| 179 | IRIS_313-10298 | G | T | T | G | T | C | 375 | IRIS_313-10809 | G | T | A | G | T | C |
| 180 | IRIS_313-10314 | G | T | T | G | T | C | 376 | IRIS_313-10815 | G | T | A | G | T | C |
| 181 | IRIS_313-10385 | G | T | T | G | T | C | 377 | IRIS_313-10816 | G | T | A | G | T | C |
| 182 | IRIS_313-10392 | G | T | T | G | T | C | 378 | IRIS_313-10817 | G | T | A | A | G | T |
| 183 | IRIS_313-11122 | G | T | T | G | T | C | 379 | IRIS_313-10827 | G | T | A | A | G | T |
| 184 | IRIS_313-11249 | G | T | T | G | T | C | 380 | IRIS_313-10828 | A | C | T | G | T | C |
| 185 | B027 | G | C | T | A | G | C | 381 | IRIS_313-10829 | G | T | A | A | G | T |
| 186 | GP628 | G | C | T | A | G | T | 382 | IRIS_313-10830 | G | T | A | A | G | T |
| 187 | IRIS_313-10045 | G | T | T | G | T | C | 383 | IRIS_313-10831 | G | T | A | A | G | T |
| 188 | IRIS_313-10109 | G | C | T | A | G | C | 384 | IRIS_313-10832 | G | T | A | A | G | T |
| 189 | IRIS_313-10441 | A | C | A | A | G | C | 385 | IRIS_313-10841 | G | T | A | A | G | T |
| 190 | IRIS_313-10448 | G | T | T | G | T | C | 386 | IRIS_313-10960 | G | T | A | A | G | T |
| 191 | IRIS_313-10519 | G | T | T | G | T | C | 387 | IRIS_313-10991 | G | T | A | A | G | T |
| 192 | IRIS_313-10525 | G | C | T | A | G | C | 388 | IRIS_313-10992 | G | T | A | A | G | T |
| 193 | IRIS_313-10542 | G | T | T | G | T | C | 389 | IRIS_313-10993 | G | T | A | A | G | T |
| 194 | IRIS_313-10547 | G | T | T | G | T | C | 390 | IRIS_313-10994 | G | T | A | A | G | T |
| 195 | IRIS_313-10554 | G | T | T | G | T | C | 391 | IRIS_313-10999 | A | C | T | G | T | C |
| 196 | IRIS_313-10576 | G | T | T | G | T | C | 392 | IRIS_313-11001 | G | T | A | A | G | T |
